# Millisecond-scale motor control precedes sensorimotor learning in Bengalese finches

**DOI:** 10.1101/2024.09.27.615500

**Published:** 2024-09-27

**Authors:** Leila May M. Pascual, Aanya Vusirikala, Ilya M. Nemenman, Samuel J. Sober, Michael Pasek

**Affiliations:** 1 Neuroscience Graduate Program, Emory University, Atlanta, United States;; 2 Department of Physics, Emory University, Atlanta, United States;; 3 Initiative in Theory and Modeling of Living Systems, Emory University, Atlanta, United States;; 4 Department of Biology, Emory University, Atlanta, United States

## Abstract

A key goal of the nervous system in young animals is to learn motor skills. Songbirds learn to sing as juveniles, providing a unique opportunity to identify the neural correlates of skill acquisition. Prior studies have shown that spike rate variability decreases during song acquisition, suggesting a transition from rate-based neural control to the millisecond-precise motor codes known to underlie adult vocal performance. By quantifying how the ensemble of spike patterns fired by cortical neurons (the “neural vocabulary”) and the relationship between spike patterns and song acoustics (the “neural code”) change during song acquisition, we quantified how vocal control changes across learning in juvenile Bengalese finches. We found that despite the expected drop in rate variability (a learning-related change in spike vocabulary), the precision of the neural code in the youngest singers is the same as in adults, with 1–2 millisecond variations in spike timing transduced into quantifiably different behaviors. In contrast, fluctuations of firing rates on longer timescales fail to affect the motor output. The consistent presence of millisecond-scale motor coding during changing levels of spike rate and behavioral variability supports the view that variability early in learning stems from deliberate motor exploration rather than imprecise motor control.

## Introduction

Motor skills learned early in life are acquired through repeated practice of motor actions until they are performed at consistently high levels. During this process of motor refinement, the brain integrates sensory feedback and relays signals from motor regions of the brain to motor actuators in the periphery to shape behavior (Arber and Costa, 2018; Krakauer et al., 2019). Little is known about the extent to which learning results from the nervous system’s refining its repertoire of spike pattern outputs (a change in “neural vocabulary”) or from changes in how the maturing body transforms muscle activity into behavior (a change in “neural code”). This is especially crucial in songbirds, where changes in both cortical spiking activity (Mooney, 1992; Ölveczky et al., 2011; Vallentin et al., 2016; Miller et al., 2017; Yuan and Bottjer, 2019; Zemel et al., 2021) and the force-producing properties of vocal muscles (Adam and Elemans, 2020; Maxwell et al., 2021) change dramatically over the period of juvenile song acquisition.

Studies that have examined how neural activity changes across learning in developing (Ölveczky et al., 2005, 2011; Avitan et al., 2020) and adult (Peters et al., 2014, 2017; Masamizu et al., 2014) animals have mostly quantified neural activity by measuring fluctuations in the spike rates of individual cortical neurons across hundreds of milliseconds prior to the onset of behavior. This approach has consistently resulted in observations of spike rate variability changing across learning. In zebra finches, the firing rate variability in motor cortical neurons was observed to drop rapidly early in the learning period, although in adulthood neurons still showed heterogeneous levels of variability (Ölveczky et al., 2011; Dhawale et al., 2017). This suggests that developmental reductions in song variability result from neurons reducing their range (vocabulary) of spike rates (Roth and Ding, 2024; Costa, 2011).

**Figure 1.**
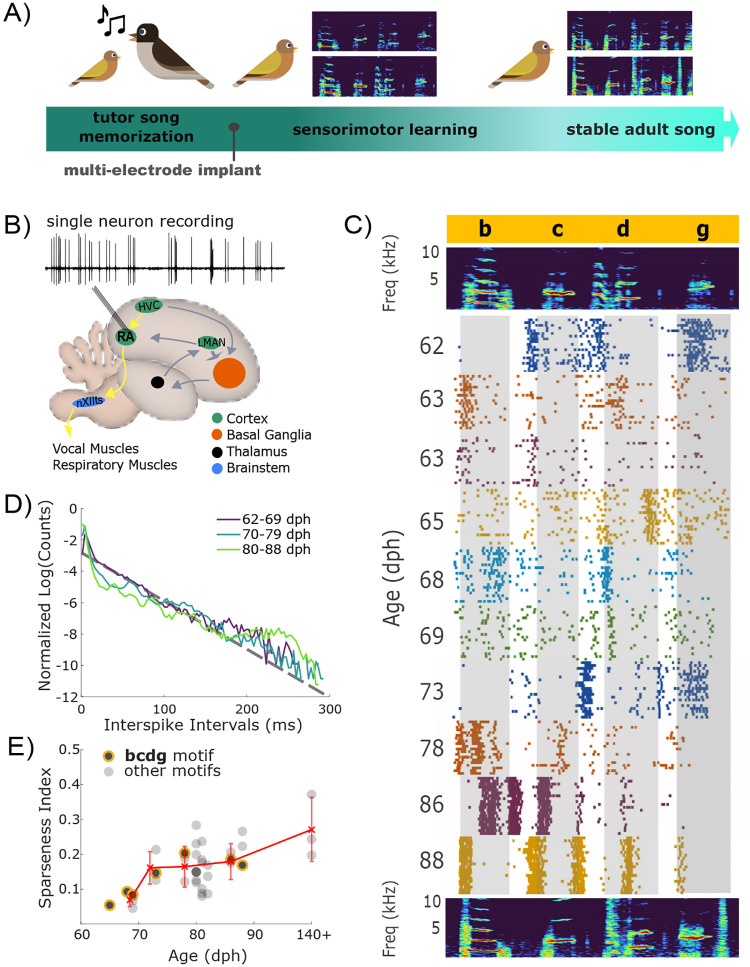
Recordings across song learning in juvenile Bengalese finches reveal changes in gross statistics of neural spiking. A) Song learning begins with early life exposure to a tutor song and continues through a period of sensorimotor practice. The two spectrograms depict a juvenile bird’s song during this period, which consists of distinct units of vocal gestures (syllables, identified with lowercase letters in Fig 1C) that are highly variable from rendition to rendition. By adulthood, the two spectrograms depict the same syllables which are highly stereotyped and closely resembling the tutor’s song. B) Spiking activity from individual projection neurons in song motor nucleus RA is recorded across sensorimotor learning. Via the song motor pathway (SMP, yellow), neurons in the motor nucleus RA send song motor commands to motoneurons in the brainstem nucleus nXIIts which directly innervate the vocal muscles. RA neurons receive input predominantly from HVC, a premotor nucleus upstream in the SMP, and from LMAN, the output nucleus of a thalamocortical basal ganglia loop which plays a necessary role in song learning (Mooney, 2009). C) The top panel is a spectrogram of a representative motif (a sequence of syllables ‘b’, ‘c’, ‘d’, and ‘g’) from a juvenile bird. The raster plots below show 20 representative spiking activities of isolated RA neurons during the song motif displayed in the spectrogram above. Each RA neuron (denoted by different colors) was recorded on a different day during song development. D) Across song learning, interspike interval distributions are reshaped from being approximately exponentially-distributed in early learning, to showing a bimodal ISI distribution, typical of bursting activity, in late learning (days are grouped in purple, blue, and green). In earlier days, the ISI distribution adhere closely to the black dashed line, which corresponds to an exponentially distributed ISI, equivalent to a Poisson model of spiking. In later days, spiking becomes severely non-Poisson, excessively spiking at small ISI values and a second mode emerging at around 250 ms. E) Sparseness index (SI) (Lehky et al., 2005; Ölveczky et al., 2011) of spiking across song learning. Black circles represent the average SI of an RA neuron recorded across iterations of a motif. Circles outlined in yellow represent SI of neurons recorded during the “bcdg” motif shown in (C). Red ‘x’ markers represent the average for different age groups.

We sought to answer how the neural motor vocabulary and code evolve across developmental learning in the juvenile Bengalese finch, a species of songbird that learns its song through a period of sensorimotor learning prior to reaching sexual maturity (~ 120 dph, Figure 1A) (Fujii et al., 2021). Across song learning, juvenile Bengalese finches develop new vocal gestures called syllables, which are acoustically refined until being “crystallized” into stereotyped sequences by adulthood (Tchernichovski et al., 2001; Okanoya, 2004; Mooney, 2022). Our previous examination of spiking variations in adult Bengalese finches revealed that the information about rendition-to-rendition variations in syllable acoustics is present at the few millisecond scale of motor cortical activity but absent in the firing rate (as measured by spike counts during a 40 ms window) (Tang et al., 2014; Hernández et al., 2022).

To examine whether millisecond variability in spike timing controls vocal motor output during the sensorimotor learning period of juvenile Bengalese finches, we recorded from neurons in vocal motor cortex – robust nucleus of the arcopallium (RA) – and examined the activity patterns within the premotor window preceding individual syllables at different developmental time points. Our analysis first shows that the spiking activity of RA neurons in juvenile Bengalese finches undergoes a major restructuring during the sensorimotor learning period, consistent with previous findings in zebra finches (Ölveczky et al., 2011). We then quantified how both the spiking vocabulary and the spiking code change across song acquisition by estimating, respectively, the entropy of each neuron’s repertoire of spiking patterns and the mutual information between spiking patterns and motor behavior. We found that despite a dramatic developmental reduction in spike rate variability (“rate vocabulary”), the vocabulary size of neurons’ precise timing patterns was nearly unchanged across behavior. Moreover, analysis of mutual information between syllable acoustics and RA spike trains at different temporal resolutions reveal a consistently precise motor code with millisecond-level structure in juvenile and adult birds. These findings suggest that throughout sensorimotor learning, vocal production is driven by precise variations in spike timing, and that the maturation of the vocal code includes the gradual abandonment of ineffective timescales of neural variability (rate variation) while preserving the component of youthful variation (precisely-timed spike patterns) that is most effective at shaping behavior.

## Results

We recorded single unit activity from RA neurons in Bengalese finches together with song acoustics across the sensorimotor learning period (62–88 dph) and into adulthood (> 120 dph) (Figure 1A). We collected data from 23 RA neurons across three juvenile birds. For one of the juvenile birds, one additional neuron was recorded after the bird reached adulthood. We combined data from this bird with adult (> 140 dph) Bengalese finch data in four birds from a previous study (Tang et al., 2014). We identified recorded neurons as putative projection neurons or interneurons based on their characteristic spike wave forms and spontaneous versus song spiking activity (see Electrophysiological recordings for details). We report data from putative projection neurons only, which comprised around 95% of our recordings as described previously (Tang et al., 2014; Sober et al., 2008).

### RA neurons undergo a major restructuring of their spike rate activity during the sensorimotor period

Studies in adult zebra finches and Bengalese finches revealed that RA spiking activity is spontaneously tonic but strongly phasic during well-learned adult song, with RA spiking characterized by temporally precise, syllable-locked bursts (Yu and Margoliash, 1996; Chi and Margoliash, 2001; Leonardo and Fee, 2005; Sober et al., 2008). While studies of RA spiking statistics in juvenile zebra finches (Ölveczky et al., 2011) suggest dramatic changes in the representation of vocal behavior across song acquisition, it is unknown whether or how the millisecond-precise spiking code described in adult Bengalese finches (Tang et al., 2014) emerges during song acquisition. Therefore, we recorded individual RA neurons from juvenile Bengalese finches across the sensorimotor learning period (Figure 1). These recordings (Figure 1 B) reveal gross changes in the statistics of firing activity across song learning (Figure 1 C). Specifically, RA neurons during early learning emit spikes with varying interspike intervals (ISIs), while later in learning spikes are emitted in more stereotyped shorter intervals characteristic of bursts, interspersed by periods of low spiking activity (Figure 1 C). In Figure 1 D, we plot the ISI distributions during song motifs (here defined as sequences of 3 successive syllables) across different juvenile age groups (62–69, 70–79, 80–88 dph). We observe that during the early stages of the sensorimotor learning period (62–69 dph), RA spiking activity is characterized by exponentially distributed ISIs, which is expected from Poisson spiking. At later stages (80–88 dph), an excess of probability at small ISI values, together with a second mode at about 250 ms are clear signatures of tonic bursting (excess of small ISIs) with large gaps between bursts (mode at 250 ms).

To characterize further the reshaping of RA activity across song learning in Bengalese finches, we computed the sparseness index of firing patterns (Lehky et al., 2005; Ölveczky et al., 2011), defined as 1 when the spiking is restricted to a single time bin during a song motif (e.g., if spikes are time-locked with a syllable onset) and 0 if spikes are evenly distributed across the motif (Figure 1 E). We found that the sparseness index increased during development from 0.069 ± 0.017 (n = 6) at 62–69 dph to 0.27 ± 0.09 in adult birds (140+ dph;n = 3). Overall, we observed a transition in activity from exponentially-distributed ISIs – typical of a Poisson spiking model – with a low sparsity in the early stages of learning to a bimodal ISI distribution and sparse activity in later stages as RA activity becomes dominated by brief, high-frequency bursts separated by epochs of silence (Yu and Margoliash, 1996; Chi and Margoliash, 2001; Leonardo and Fee, 2005; Sober et al., 2008).

### Variability of spike rate and song acoustic features of individual syllables show an overall decrease during learning

**Figure 2.**
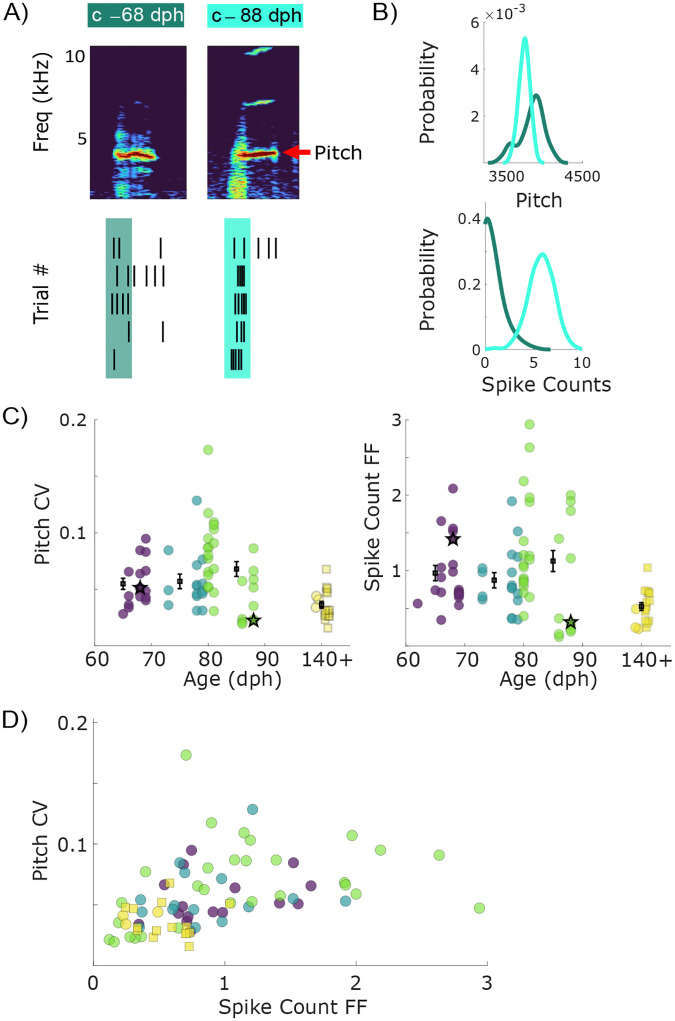
Variability of syllable acoustics and spike count change drastically during song learning. A) Representative spectrograms of syllable ‘c’ during early learning (68 dph) and late learning (88 dph) reveal changes in acoustic structure, which we quantify across learning (e. g., as changes in the fundamental frequency, or pitch). Similarly, changes in RA spiking activity during the 40 ms premotor window (shaded) are quantified across learning. B) The coefficients of variation in the pitch distributions of syllable ‘c’ decrease from 68 dph (light blue, CV = 0.051) to 88 dph (darker blue, CV = 0.022). Spike count variability concomitantly decreases from 68 dph (Fano Factor = 1.42) to 88 dph (FF = 0.32). C) Pitch CV and spike count Fano factor for all analyzed syllables are depicted by individual circles. Values for the previously reported data (Sober et al., 2008; Tang et al., 2014) are shown by a square. The error bars show the mean ± SEM for each age group. Syllable ‘c’ on 68 dph and 88 dph is depicted with stars. D) Variability in pitch correlates positively with variability in spike counts. Syllables from more mature birds are in the lower left quadrant, indicating decreased pitch and spike count variability compared with syllables from younger animals.

The observed global restructuring of spiking activity across learning warrants further exploration of the corresponding changes in both the vocabulary of motor commands (that is, which spike patterns are used by RA neurons) and the neural code (how these patterns are transduced into motor behaviors). We thus begin by characterizing the variability of individual neurons’ spiking vocabulary – namely, the total spike count in the premotor window (Figure 2 A) – and relate its change with the changes in variability in acoustic features of individual song syllables during learning.

As can be seen in Figure 2 B, we observed a reduction in acoustic variability as evidenced by the narrowing of the pitch distribution for individual syllables with increasing age, and quantified by a corresponding decrease in the coefficient of variation (CV) of the pitch distribution (Figure 2 C). Across all syllables and ages, the pitch distributions of individual syllables from juvenile birds (62–90 dph) showed wide ranging levels of variability with incidences of syllables with high variability values (CV > 0.1), while pitch variability was significantly smaller in adult birds.

As a simple analysis of the changes in the RA neural vocabulary corresponding to individual syllables, we measured the number of spikes occurring in a 40 ms “premotor window” before the time, at which the acoustic properties of each syllable were measured (Figure 2 A). Trial-to-trial variations in this premotor activity were previously shown to correlate with variations in acoustic structure of individual syllables in adult birds (Sober et al., 2008). We observed a relative narrowing of the spike count probability distributions with increasing age (Figure 2 B), as quantified by a decrease in the Fano factor (FF) of spike counts (Figure 2 C). Furthermore, we found that variability in pitch of individual syllables positively correlated with spike count variability of premotor activity (Figure 2 D). The presence of syllables with high trial-to-trial variability in spike counts and acoustic features early in the sensorimotor learning period raises the possibility that trial-to-trial variations in acoustics are encoded by spike counts (Shadlen and Newsome, 1998; Brette, 2015). However, this analysis cannot distinguish if the overall decrease in trial-to-trial spike count variability and the associated acoustic variability during sensorimotor learning reflects an increase in the precision of the motor code. Alternatively, this decrease can be caused by a reduction in the vocabulary of motor commands at a fixed precision of the motor code.

To make a first clarifying step towards distinguishing the possibilities, we turn next to a finer characterization of the vocabulary of motor commands being used at different time points during the sensorimotor learning period by using information theoretic measures.

### Variability in millisecond-scale activity patterns in juvenile birds remains constant across learning

**Figure 3.**
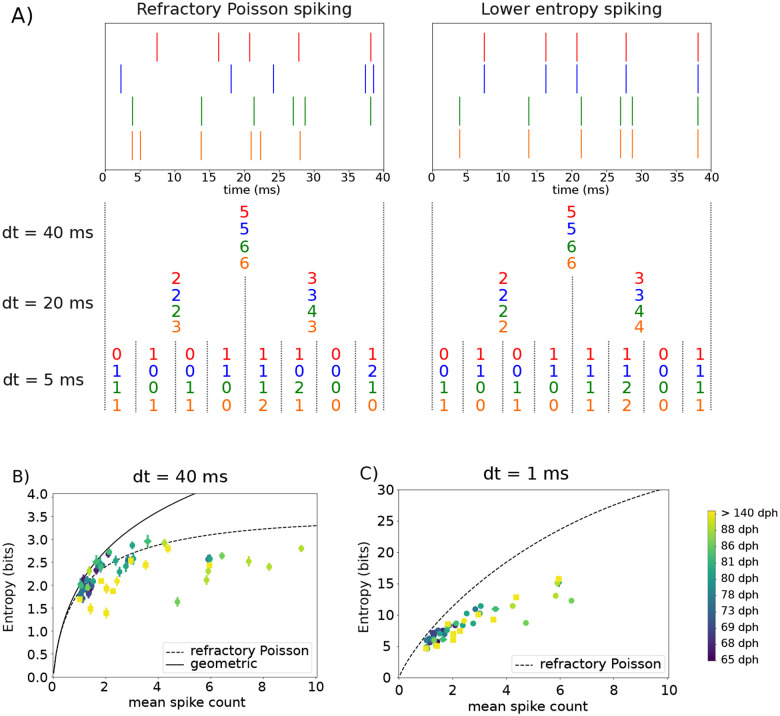
Entropy of RA activity at high temporal resolution suggests a non-Poisson neural timing code with statistical properties similar across days. A) Schematic for the representation of a 40 ms spike train discretized at different temporal resolutions *dt*. Each spike train represents a different trial of the activity of a given RA neuron during the 40 ms premotor window (see Text). Each number in the representation corresponds to the number of spikes in a specific bin of size *dt*. Left panel: four different spike trains from a refractory Poisson process. Observe that different spike trains can have the same representation at *dt* = 40 ms but different representations at higher temporal resolutions, e.g., dt = 5 ms. Intuitively, having different representations for different trials leads to a larger vocabulary at this resolution, and hence to a high value of entropy. In contrast, having the same representation across different trials leads to a smaller vocabulary and a lower value of entropy. Right panel: A neuron with reproducible spike patterns would have the same entropy as the refractory Poisson process at *dt* = 40 ms, but a lower entropy at higher temporal resolution. B) and C) Entropy of RA spike trains at different temporal resolutions as a function of the mean spike count during the premotor window. Entropy was calculated using the NSB method (see Entropy and mutual information estimation) at discretization of 40 ms for (B) and 1 ms for (C). Note that *dt* = 40 ms is equivalent to analyzing the overall spike count in the entire premotor window. Each neuron/day of recording is represented by a different color, from dark blue (age of 65 dph) to yellow (age > 140 dph), see color bar. We plot for comparison the entropy curve for a refractory Poisson process with the refractory period of *τ* = 1 ms, and the entropy of the geometric distribution of spike counts (the latter for *dt* = 40 ms only).

Although we observed an overall decrease in the firing rate variability of individual syllables during the sensorimotor learning period, characterizing a change in the vocabulary of motor commands requires us to quantify the variability present at different timescales of RA activity. A suitable measure of variability is the entropy of the ensemble of spike trains – which can be discretized at different temporal resolutions – corresponding to the different renditions of a given motor output (Strong et al., 1998; Rieke et al., 1999) (see Entropy and mutual information estimation for the definition of entropy and details on its estimation). The resulting discretized activity for each rendition can be thought of as a spike “word” formed by a sequence of symbols, where each symbol represents the number of observed spikes during each time bin of size *dt* (see Figure 3 A). The ensemble of such spike words for each individual syllable is then a representation of the corresponding vocabulary of motor commands with a time resolution *dt*.

Two spike patterns that differ in their precise spike timing may be identical when discretized at a longer timescale (see Figure 3 A). Thus, in Figure 3 B–C, we plot the entropy of spike words for individual syllables recorded at different developmental time points analyzed at two different timescales, *dt* = 40 ms (Figure 3 B) and *dt* = 1 ms (Figure 3 C). In both cases, we analyze the entropy as a function of the mean spike count during the premotor window, and we compare the entropy of the recorded vocabulary with the entropy of a refractory Poisson process with a refractory period *τ* = 1 ms, as well with the entropy of the geometric distribution (see Quantification of neural variability for details). When spike patterns are analyzed at the scale of the whole premotor window (*dt* = 40 ms), we observe that the entropy of spike words in both adults (> 120 dph) and juveniles (65–88 dph) follows relatively closely the entropy of a refractory Poisson process as the mean spike count increases (Figure 3 B). Additionally, we found that vocabularies in adults (> 140 dph) tend to have lower entropy values than in juveniles at similar mean spike count (Figure 3 B), confirming our findings that juvenile syllables show a higher degree of variability in trial-to-trial spike count values compared to adults. Although we observe some juvenile cases lying above the refractory Poisson entropy values, no adult syllable was found to show a similar high-entropy, “super-Poisson” behavior. Crucially, all of the high entropy values were found to lie below the entropy of the geometric distribution. This is a crucial consistency check since the latter is the maximum entropy distribution at a fixed mean count (Rieke et al., 1999).

To investigate the level of variability of motor commands at a different, smaller temporal resolution, in Figure 3 C, we plot the entropy of RA spike patterns discretized at a smaller resolution of *dt* = 1 ms, with a total word length set to 40 ms (the length of the premotor window) as above. The entropy values of spike patterns binned at *dt* = 1 ms are notably larger than those at *dt* = 40 ms (Figure 3 B). However, this is expected from the corresponding increase in the number of all possible spike words in this case. More interestingly, all entropy of vocabularies lie significantly below the entropy of a refractory Poisson spike train analyzed at the same resolution of *dt* = 1 ms, irrespective of age.

Our finding that the entropy vocabularies in RA for both juvenile and adult birds at 1 ms resolution is significantly lower than expected from the variability observed at coarser resolution suggests that the vocabularies have a nontrivial temporal structure. A schematic representation of this structure can be seen in Figure 3 A. We observed that the entropy of the vocabulary at *dt* = 40 ms resolution in both juveniles and adults is roughly compatible with refractory Poisson spiking. One would thus expect on the basis of this finding that the raster plot for four different renditions of a given syllable would look somewhat like the left panel of Figure 3 A, which was generated from a refractory Poisson process. However, such relatively structureless, “noisy” activity patterns would also yield entropy values compatible with a refractory Poisson process when binned at smaller time resolutions. Instead, we found that the entropy of vocabularies is significantly below the entropy of a refractory Poisson spike train when analyzed at *dt* = 1 ms. This suggests the presence of a much reduced vocabulary of motor commands as compared to the one expected from the noisy Poisson process, with the existence of over-represented millisecond-scale codewords (right panel of Figure 3 A) irrespective of the time of recording during the sensorimotor learning period. This is the first indication that the large vocabulary of motor commands early in learning is not noise-dominated but may reflect the exploration of motor commands.

This analysis only explores the vocabulary of spiking, but leaves the question of the reliability of the neural code still open: how reliably are different motor commands mapped into different motor outputs? Answering this question is equivalent to answering what fraction of the variability in neural activity can be explained by taking into account the variability in the motor output. One can answer this by measuring the mutual information between syllable acoustics and RA activity patterns at different timescales, to which we now turn.

### Acoustic variability of individual syllables is encoded by millisecond timescale variations of RA activity over the entire duration of learning

The different degrees of variability found in the motor commands at different resolutions of RA activity raise the question of which part of this variability is used by the motor code to transmit information about the motor output. To answer this question, we estimated the mutual information between the activity patterns at different temporal resolutions and various parameters characterizing the vocal output. This parallels the analysis of Tang et al. (2014), but now we also estimate the mutual information as a function of the bird’s age. As in Tang et al. (2014), for three different such parameters – pitch, amplitude, and spectral entropy – we assigned every rendition of each song syllable to group 1 (resp. 2) if the syllable rendition was lower (resp. higher) than the median value of the acoustic parameter for all iterations of this syllable.

**Figure 4.**
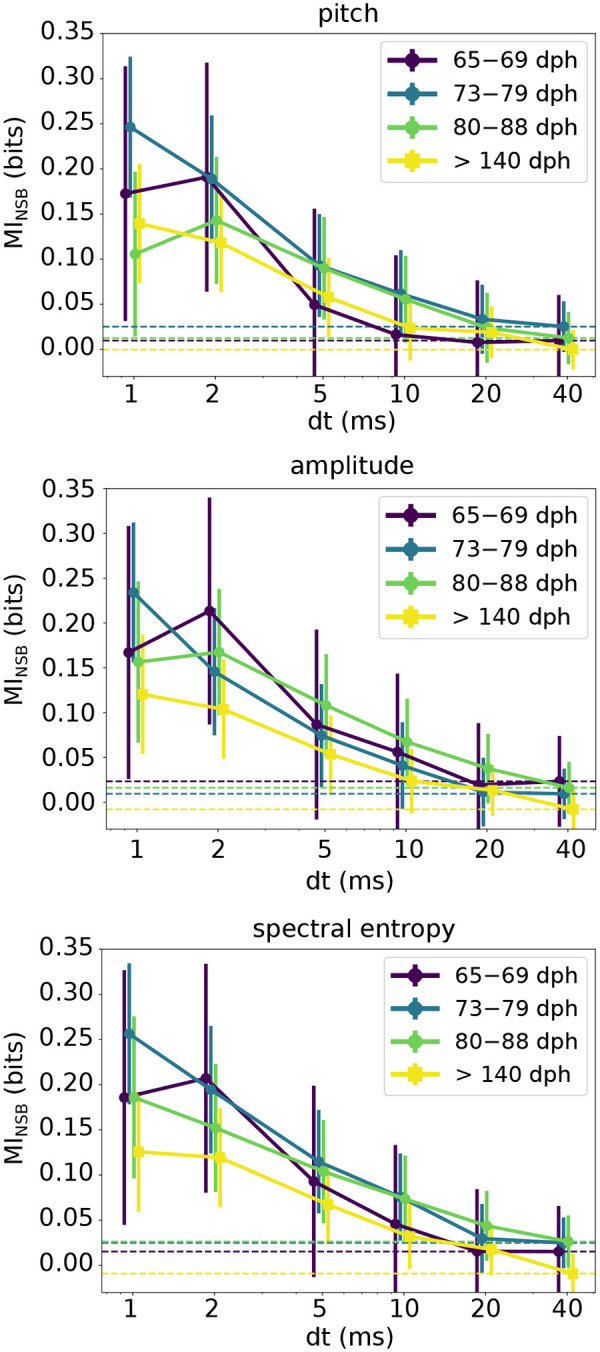
A precise timing code for vocal behavior control is observed in both juvenile and adult birds. Mutual information between the spike words and acoustic features at different temporal resolutions *dt*, as estimated by the NSB estimator (see Entropy and mutual information estimation). The three panels display values of mutual information between the spike train and three acoustic features: a) pitch, b) amplitude, and c) spectral entropy. Error bars represent one standard deviation (SD) of the information estimate, evaluated as in Entropy and mutual information estimation). We find that, in all three cases, and across all age categories, from early juvenile (65–69 dph, and 73–79 dph), to late juvenile (80–88 dph), and to adult, the mutual information increases as *dt* decreases, at least to about 2 ms. Additionally, the mutual information at the spike count scale, *dt* = 40 ms, is not statistically different from zero, indicating that the spike rate does not have a measurable effect on the studied acoustic features. Adult neural and acoustic data are from Tang et al. (2014), which we reanalyzed. Note that we consider two acoustic groups for each acoustic feature, thus the maximum possible shared information is one bit.

In Figure 4, we plot the mutual information estimates averaged over different syllables and different days in four different age categories (65–69 dph, 73–79 dph, 80–88 dph and > 140 dph), see Entropy and mutual information estimation for details. We found that the mutual information between the neural activity patterns and the behavioral group describing trial-to-trial variations of syllable acoustics strongly depends on the resolution at which the neural pattern is analyzed, as was found previously in adult birds (Tang et al., 2014). Moreover, we found that variations in the pattern of activity of single RA neurons on timescales greater than 10 ms are not predictive of trial-to-trial variations of a syllable in any of the acoustic parameters (pitch, amplitude and entropy) that we analyzed. We also found that, surprisingly, the amount of information at the 1–2 ms timescale is similar for all age categories (Figure 4). This points towards the possibility that the encoding of fine behavioral variations by ~ 1 ms fluctuations of spike timing of RA activity precedes the late stages of the sensorimotor learning period. This finding stands in stark contrast with expectations that the behavioral variability seen in juveniles stems from an as yet imprecise transformation of the neural activity into the motor output.

**Figure 5.**
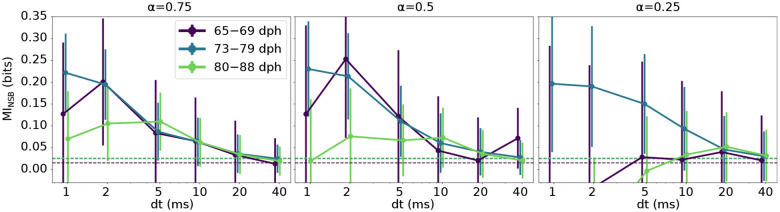
Behavior of the mutual information as a function of the data set size supports the hypothesis of the temporally precise neural code. We plot mutual information estimates as a function of the discretization scale *dt* of RA activity patterns and the sample set size for different age categories. Each panel correspond to a different fraction *α* of the full dataset of size *N* used for mutual information estimation. a) *α* = 0.75, b) *α* = 0.5, c) *α* = 0.25. Below *α* = 0.5, mutual information at high temporal resolution is statistically indistinguishable from zero, suggesting that the evidence of temporally precise neural code in Figure 4 is not an artifact of sample-size dependent estimation biases.

In our analysis, mutual information between the spike train and the acoustic features is a small difference between two large entropies: that of the vocabulary and that of the vocabulary conditioned on a behavioral group. Thus, relatively small biases in estimation of each of these entropies may result in large mutual information biases. While we worked hard to remove such biases, cf. Entropy and mutual information estimation, it is crucial to provide an independent assessment of the quality of the mutual information estimation used here. For this, we note that all entropy (and hence mutual information) estimation biases are sample-size dependent Paninski (2003), typically decreasing polynomially as the sample size increases. Thus, if the increase of the mutual information at high temporal resolution in Figure 4 is due to residual estimation bias, we would expect the values to be larger for smaller sample sizes. To verify this, we repeated the mutual information estimation for different fractions of our total dataset (Figure 5). We observe that our main finding from Figure 4 – namely that information about trial-to-trial variations of individual syllables is encoded at the 1–2 ms resolution throughout the sensorimotor learning period – disappears when the dataset size decreases by 50%. This supports the assertion that the increase of mutual information at small *dt* is not due to the estimation bias, but is a bona fide feature of the system.

## Discussion

In this study, we examined the changes in the spiking vocabulary emitted by single neurons in RA across skill learning during development, and we investigated the origins of millisecond-scale control of vocal behavior in adult songbirds. Our results show that underlying the dramatic transformations in cortical spiking vocabulary throughout vocal learning (Figure 1) is a millisecond-scale motor coding scheme that exists even prior to the onset of the sensorimotor learning phase in juvenile songbirds (Figure 4). While the neural code itself can change during learning, its temporal precision is stable. Furthermore, we reveal that RA neurons use a sub-Poisson spiking vocabulary, and this does not change significantly into adulthood. This suggests that the motor system can transduce the neural commands into vocal behavior reliably as early as the beginning of the vocal learning period.

This contradicts a common lore in the field that RA neurons can “afford” to be noisy early in learning since the subsequent mapping of the neural activity into the vocal behavior is also imprecise. Then, later in learning, as the motor system matures, RA neurons must develop more regular activity patterns as well. Instead, our results suggest that the motor system can use millisecond-scale variations in spike timing to control the acoustics of individual syllables consistently for all examined ages. This provides evidence for the existence of a motor learning system in the juvenile songbird that is equipped to undergo a process of motor exploration: the behavioral outcome of each spike word is narrowly distributed, and hence it provides a meaningful sensorimotor feedback that enables evaluation and choosing of ethologically-relevant spiking vocabularies for learning the target song.

A model of sensorimotor learning has been proposed in which a period of exploring a large neural activity space leads to the selection of specific neural patterns that enact favorable behavioral outcomes (Dhawale et al., 2017; Costa, 2011). A spike timing code would support a rapid selection of favorable neural patterns, a process possibly reflected by the dramatic transformation in RA spiking statistics across learning (Figure 1 C–E): we observed increased sparseness and a gradual shift in the inter-spike interval distributions towards higher occurrences of bursts. These changes are likely influenced by shifts in the sources and characteristics of inputs into RA over the course of song learning. Previous work in juvenile zebra finches which reported similar trends in RA spiking across development (Ölveczky et al., 2011) also showed that the inhibition of HVC and LMAN (the two predominant sources of input to RA) led to a reduction in RA spike bursts, thus potentially implicating external inputs, at least partially, for the increase in RA burst firing during learning. As the balance of inputs to RA shifts away from LMAN and in favor of HVC across song learning (Mooney, 1992; Stark and Perkel, 1999; Mehaffey and Doupe, 2015; Morrison and Nottebohm, 1993; Ölveczky et al., 2005; Boettiger and Doupe, 2001; Palmer et al., 2021; Roberts et al., 2010; Tupikov and Jin, 2021), changes in HVC neural activity could play an increasingly pronounced role in shaping RA spiking. Indeed, it was shown that during learning, HVC neurons projections decrease the intervals between spikes in bursts and also decrease the presence of single spikes emitted outside of bursts (Bistere et al., 2024). Furthermore, HVC projection neurons were also observed to decrease in activity during well-learned syllables due to increased activation of HVC interneurons (Vallentin et al., 2016).

In addition to inputs extrinsic to RA, fast-spiking inhibitory interneurons within RA might also contribute to changes in RA spiking. Across song learning, unidirectional interneuron-to-projection neuron connections decrease while bidirectional connections are preserved (Miller et al., 2017). This shift in favor of reciprocal connections between interneurons and projection neurons could play a role in the increased sparseness in RA spiking across learning and, importantly, in shaping syllable acoustics. Furthermore, fast-spiking interneurons in RA demonstrate properties (e.g., narrow spike width, short after-hyperpolarization duration, and non-adapting spike trains) which give them unique capabilities of shaping projection neuron spike timing activity with high (millisecond to sub-millisecond) temporal resolution (Miller et al., 2017).

Our results showing concomitant changes in syllable acoustics and spike count variability (Figure 2) are consistent with prior work on the role of behavioral and neural activity variability in learning (Dhawale et al., 2017; Costa, 2011). However, when we examined RA spiking at higher temporal resolution (*dt* = 1 ms), we found no differences in the entropy of RA spiking vocabulary for syllables from juvenile birds compared to that of adult birds (Figure 3), with entropy values for both deviating similarly from values expected for a refractory Poisson process. Therefore, although RA neurons vary their spike rates widely during learning, RA neurons seem to use a restricted spiking vocabulary. This suggests a learning strategy that is perhaps promiscuous in exploiting a large area of the neural space, yet selective in using specific neural patterns.

We emphasize again the main conclusion of our work. Analysis of neural and behavioral variability does not, by itself, resolve multiple possibilities about the mapping of neural activity to behavior. Two extreme possibilities can account for the reduction of the variability: 1) spike patterns encode behavior poorly at the start of learning and improve across learning, or 2) spike patterns encode behavior with the same accuracy from the start of learning. Our results demonstrating that RA neurons already use a millisecond spike timing code at the earliest age group that we recorded during development suggests that the precise coding scheme precedes the start of sensorimotor learning, so that the second hypothesis is closer to the truth. Prior work examining the activation dynamics of songbird vocal muscles has shown that superfast muscles exist in the songbird syrinx prior to sensorimotor learning (Adam and Elemans, 2020). In juvenile zebra finches, the acoustic output of the sound generators of the syrinx do not change considerably over postnatal vocal development, suggesting that the properties of the vocal apparatus are not changing, but rather the control of the apparatus does (Maxwell et al., 2021). The coding scheme of signals relayed from cortex to impact vocal acoustics, therefore, need not change across learning. Merely changing the vocabulary of motor commands can achieve the learning goals. Crucially, here we only showed that the precision of the neural code—but not necessarily the code itself—is invariant during learning. Detecting specific codewords, as in Hernández et al. (2022), and analyzing their behavioral consequences across learning is essential for tracking the evolution of the code itself. This would require an ability to record spiking activity from the same neuron for days at a time. While this is still beyond reach in the songbird system, the existence of a precise code even early in learning, which we showed here, adds urgency to developing the necessary technology.

## Methods and Materials

### Subjects

Male Bengalese finches (*Lonchura striata var. Domestica*) were bred in our colony and housed with their parents until 60 days of age. After electrode implantation, birds were isolated and housed individually in sound-attenuating chambers with food and water provided *ad libitum*. All recordings are from undirected song (i.e., no female was present). Recordings from three juvenile birds (60–100 dph) were collected using a previously described experimental protocol (Sober et al., 2008). A subset of the recordings from three adult birds were collected from the same juvenile birds that had matured into adulthood. The remaining subset of recordings from four adult birds (>140 dph) were previously collected as part of a separate analysis (Sober et al., 2008). Procedures were performed in accordance with established animal care protocols approved by the Emory University Institutional Animal Care and Use Committee.

### Song Acoustic Analysis

#### Song Detection

A microphone placed inside the housing chamber chronically recorded song simultaneously with neural recordings. To quantify acoustics of song syllables, we first detected the presence and identity of song syllables from the audio recordings. Syllable onset and offset times were determined on the basis of amplitude threshold crossings after smoothing the acoustic waveform with a square filter of width 2 ms. The error of our determination of syllable onset time is therefore on the order of milliseconds. This millisecond-scale uncertainty in syllable onset time cannot account for our results since millisecond-scale jitter would decrease, rather than increase, our information estimates at fine timescales.

#### Syllable Acoustic Quantification

The identities of song syllables were determined by visual examination of spectrogram renderings of song behavior. For each syllable identity, we determined a particular time (relative to syllable onsets) when the spectral features were well defined to quantify vocal acoustics. This time of quantification was taken to be fixed relative to the syllable onset, i.e. it does not vary between different iterations of a given syllable. We analyzed the acoustic power spectrum at the specified time during each iteration of a song syllable and quantified the fundamental frequency (which we refer to as “pitch”), amplitude, and spectral entropy. We chose to quantify these three acoustic features since they capture a large percentage of acoustic variability in Bengalese finch song and are the features that are refined during song learning (Sober et al., 2008).

### Electrophysiological recordings

#### Surgical Implantation of Microdrive

Birds were anesthetized (induction with 3% isoflurane, maintained with 1.5–2% isoflurane) and a lightweight 16-microelectrode bundle microdrive (Innovative Neurophysiology) was stereotactically positioned above RA nucleus in one hemisphere (2 implants over right RA, 1 over left RA) and secured to the skull with dental cement. After birds recovered from surgery and singing resumed (within 1–3 days), electrodes were advanced through RA using a miniaturized microdrive which recorded extracellular voltage traces during and between bouts of singing. RA recording sites were confirmed by the presence of characteristic changes in activity associated with the production of song and calls and by *post hoc* histological confirmation of electrode tracks passing through the RA nucleus.

#### RA Unit Isolation

To isolate the spiking activity from individual units, we used a previously-described spike sorting algorithm (Sober et al., 2008), which provided a scalar measurement of unit isolation to establish a quantitative-based inclusion criterion for our spike train analyses. Briefly, we determined a voltage threshold to detect both spike and noise waveforms from singing-related activity. We then performed principal components analysis (PCA) on these waveforms and used the first two principal components to project a 2-dimensional representation of the waveforms. We then assigned each waveform to a cluster by applying an automated nearest-neighbor clustering algorithm (kmeans.m in MATLAB, The MathWorks, MA) to the 2-dimensional data. For the majority of cases, two clusters were selected: a “spike” and a “noise” cluster.

We obtained a measure of a unit’s isolation by quantifying the extent of overlap between the spike and noise clusters. To do this, we fit a 2-D Gaussian to each cluster to estimate the mean and variance of each cluster. We then used the Gaussian fits to generate 10,000 synthetic points for each respective cluster. We re-applied the nearest-neighboring algorithm on the synthetic points and quantified the extent of cluster overlap by the frequency, with which synthetic points were miscategorized by the algorithm. We deemed that a frequency of less than 0.01 of cluster overlap (or “isolation error”) was a reasonable threshold for classifying the spike cluster as a single-unit.

#### Unit Inclusion Criteria

We examined the interspike interval (ISI) distributions of each successfully isolated single-unit. Only single units with < 1% of interspike intervals less than 1 millisecond were included in our analysis or RA activity from juvenile birds. Based on prior characterizations cell-type specific spike waveform shape and response properties (Sober et al., 2008), RA recordings were classified as putative excitatory projection neurons or interneurons. Only RA projection neurons were included in the analysis for this study. In total, we collected 30 RA single-unit recordings. Recordings spanned 27 days across song learning for Bird 1, 5 days for Bird 2, and 3 days for Bird 3.

### Spiketrain analysis

#### Entropy and mutual information estimation

To quantify the amount of information on vocal acoustics that is conveyed by the activity of RA neurons at different timescales, we estimated the mutual information Shannon (1948) between discretized measures of neural activity within the premotor window and the “behavioral group” of syllables, based on acoustic features of iterations of individual song syllables or hand-labelling syllable identities across different syllables. We did this at different temporal resolutions of the neural activity, as is commonly done (Strong et al., 1998; Nemenman et al., 2004; Tang et al., 2014). As in previous analyses (Tang et al., 2014; Hernández et al., 2022), the premotor window was defined relative to the time of quantification for acoustic features and taken to be of length *T* = 40 ms.

Within the premotor window, we discretize the spiketrain into bins of duration *dt*, with 1 ≤ *dt* ≤ 40 ms. We then digitize the spiketimes, i.e. set the value in each time bin to the number of spikes in that bin. Given the existence of a refractory period $\tau_{\text{ref}}$ in RA neurons of approximately 2 ms (Miller et al., 2017; Zemel et al., 2021), we do not expect more than one spike to be present per 1 ms bin, but larger *dt* can result in more than one spike per bin. Thus, the spike times in each premotor window are translated into a spike word containing *T / dt* digits, with each digit equal to the spike count observed within each time bin of size *dt*.

To analyze the properties of the neural code at different timescales, we perform our mutual information analysis at different time resolutions *dt* = {1, 2, 5, 10, 20, 40} ms (these values fit into the premotor window *T* = 40 ms integer number of times, and they are roughly uniformly spread when plotted on a log-scale axis).

Using these $N_{\text{trials}}$ spike words R of size *T* / *dt*, together with the $N_{\text{trials}}$ acoustic group indices G for each acoustic feature, one can then estimate the mutual information between the spike train and the motor output through a difference in two entropies (Tang et al., 2014)

(1) $$I_{T,dt}(R;G)=H_{T,dt}(R)-{\left\langle H_{T,dt}(R|G)\right\rangle }_{G},$$

where $\langle \ldots \rangle_{G}$ represents an average over the behavioral group G. The mutual information Equation 1 quantifies the reduction in uncertainty of a random variable R, as measured by the entropy H(R), due to the knowledge of another random variable G, as quantified by the conditional entropy H(R∣G) (Cover and Thomas, 2012).

Given a probability distribution p(R), the entropy H(R) is defined as

(2) $$H(R)=-\sum_{R}p(R){\text{log}}_{2}p(R).$$

Obtaining an accurate estimate of the entropy from finite datasets is a notoriously difficult problem (Paninski, 2003), as it requires an estimation of the probability distribution p(R) from a finite number of samples. In the asymptotic sampling regime, where each response R has been sampled multiple times, the “naive” or “maximum likelihood” estimate can be used, which replaces probabilities by empirically observed word frequencies $p(R)\approx f(R)=n_{R}/N$ (where $n_{R}$ is the number of times a specific response R was observed, and N is the total number of different responses) in the definition of the entropy Equation 2. Such an estimate of the entropy is biased, with the bias converging to zero as N→∞ (Paninski, 2003). The convergence is slow, ~|R|/N, where |R| is the cardinality of the variable, whose entropy is being analyzed. However, often one is interested in estimating the entropy for distributions over the space of possible responses with a very large cardinality. The cardinality of possible responses depends exponentially on the size of the time window T and the time binning resolution *dt*, and can, in principle, be very large, precluding the use of the naive estimator.

Indeed, we estimate the cardinality $|R{|}_{\text{est}}$ of the observed spike trains using the standard estimate for the number of “typical” sequences when drawing n independent and identically distributed discrete random variables (known as the “asymptotic equipartition property”) (Cover and Thomas, 2012):

(3) $$|R{|}_{\text{est}}=2^{nH_{\text{est}}(R)},$$

where n=T/dt is the number of bins used to discretize the spike trains, and we take the entropy estimate $H_{\text{est}}(R)$ to be the entropy of a Poisson distributed counts for the number of spikes in each bin with mean $\lambda =\left(\left\langle n_{\text{sp}}\right\rangle +3\sqrt{\left\langle n_{\text{sp}}^{2}\right\rangle -{\left\langle n_{\text{sp}}\right\rangle }^{2}}\right)dt/T$, where $n_{\text{sp}}$ is the total number of spikes observed during the premotor window T in each trial, and ⟨…⟩ represents an average over trials. At the finest discretization considered here, *dt* = 1 ms, and for a typical average spike count, we find the number of possible neural words is on the order of $|R{|}_{\text{est}}\sim 10^{10}$. As this cardinality is much higher than the typical number of trials per group (n≳200) that we were able to collect in our data, we face the task of giving a reliable estimate of entropy in the severely undersampled regime.

Because of this constraint, we use the Nemenman-Shafee-Bialek (NSB) entropy estimation technique (Nemenman et al., 2001; Nemenman, 2011; Hernández et al., 2023). This is a Bayesian approach that provides an estimate of the entropy, together with an error bar on the estimate that is the standard deviation of the posterior distribution. It has no systematic bias for short-tailed distributions (Hernández et al., 2023) – in contrast to the maximum-likelihood estimate – even in the strongly undersampled regime. The ability of the NSB approach to give an unbiased entropy estimate in the undersampled regime lies in its dependence on counting the number of “coincidences” in the dataset, i.e., the number of times each spike “word” is seen more than once over repeated trials (Nemenman, 2011). As a reliable entropy estimate can be provided by the NSB approach only if the number of coincidences in the data is significantly greater than one, we verified that this criterion is satisfied for the observed word distributions in all syllables and acoustic groups. We found that the number of coincidences in the data was high enough to yield a reliable estimate of entropy when the minimum number of trials for each individual syllable was greater than 200. Only these syllables were included in our analysis. Additionally, we restricted our analysis to syllables for which mean spike count values were greater than one, as this corresponds to our threshold for defining an RA unit as “active” (Sober et al., 2008). A total of 46 syllables across 3 birds and ages from 65 dph to 153 dph satisfied this criterion.

The NSB approach to estimating the entropy relies on having a fast (e.g., exponential) decay of the rank-ordered distribution of words as a function of the rank (Nemenman, 2011; Hernández et al., 2023) (see also (Camaglia et al., 2024)). In particular, entropy estimates for rank-ordered distributions of words with long (e.g., power law) tails tend to underestimate the true entropy (Nemenman et al., 2004, 2008). To circumvent this problem, we make use of an exact relation for the additivity of entropy for a mixture of two disjoint partitions of data (i.e., containing no overlapping words), see Appendix 1 for a derivation of the formula for the additivity of entropy and the corresponding error bars on the total estimate. Thus, similarly to Nemenman et al. (2008); Tang et al. (2014), we partitioned trials into the most common word vs. all other words in cases (i.e. for a given syllable on a given day) where the most common spike word in the distribution appeared in more than 2% of the recorded trials. For juvenile birds, the most common word is in all cases the “silence” word with no spikes.

To find out if our entropy estimates are indeed free from any statistical bias given the limited dataset sizes we could reach in our experiments, we performed a series of tests, to verify that: firstly, temporal correlations across different trials are low and the different spike words can be assumed to be independent samples of the underlying word distribution, and secondly, that our dataset does not suffer from undersampling bias. We give a short description of these two tests below, following Tang et al. (2014), while the results of these tests can be found in Appendix 2.

The absence of significant temporal correlations in the spiking data for every syllable/neuron in the course of a single day was assessed by comparing the difference in entropy estimates between the first and second half of all trials, with the difference in entropy estimates between even and odd trials. The latter is taken as a reference, where any contribution from long time correlation effects should be cancelled out. As these two differences in entropy estimates were comparable, we ruled out the presence of significant temporal correlations over the course of each day.

To address the potential issue of finite sample bias in our data, we verified that individual conditional and unconditional entropy estimates showed little variations when computed from smaller data fractions than our complete dataset (Strong et al., 1998; Nemenman et al., 2008; Tang et al., 2014). More specifically, when estimating the entropy for a case with N number of trials recorded, we also estimated the entropy H(α) for αN, with α<1, randomly selected trials and averaged over 10 realizations of this subsampling to yield the subsampled-average entropy estimate ⟨H(α)⟩. By observing the finite data size scaling behavior of ⟨H(α)⟩ as a function of the inverse data fraction 1/α→1, we could confirm that a large fraction of our entropy estimates were in an asymptotic regime of sufficient data size.

To obtain the mutual information curves for each age range in Figure 4, we averaged the mutual information estimate Equation 1, first over all cases recorded in each day, and then over the different recorded days in each age range. The averaging in both cases was performed by “inversevariance weighting”, i.e., by weighing the entropy contribution of each case by the inverse of the posterior variance of its estimate, as in (Tang et al., 2014). The error bar on the mutual information average for each age range in Figure 4 represents the standard deviation of the mean, and was similarly calculated by averaging the individual posterior variance of each estimate, first for all cases in each day, and then over the different recorded days in each age range. These were again weighed by the inverse of the posterior variance of the corresponding estimate. To further confirm the absence of undersampling bias in our data, we repeated the data fraction analysis described above on the final averaged mutual information estimates. We observe that our main finding from Figure 4 – namely that mutual information between spike timing and behavioral variation within a given syllable is higher at finer resolution, *dt* = 1 ms – is preserved when we subsample our dataset down to 50% of its full size, see Appendix 2 and Figure 5.

#### Quantification of neural variability

A standard way of estimating the variability of spike trains is to calculate the Fano factor (Gerstner et al., 2014)

(4) $$F=\frac{\left\langle {\left(\Delta n_{\text{sp}}\right)}^{2}\right\rangle }{\left\langle n_{\text{sp}}\right\rangle },$$

which allows to compare the variability of spike trains with different mean firing rates $\left\langle n_{\text{sp}}\right\rangle /T$. If the distribution of spike counts is modeled as an homogeneous Poisson process, where random occurrences of spikes are independent and happen with a constant rate, the Fano factor is exactly one. This allows to gauge neural variability by measuring its distance to Poisson-like firing. However, the Fano factor gives an estimate of the variability only at the relatively coarse scale of the measured spike count during time window T. Additionally, RA neurons have been observed to display a refractory period (which forbids mutiple spikes from happening in close succession), a fact that is not taken into account by the simple Poisson process.

To quantify the variability in spiking activity at different scales, including millisecond timescale, and different time points during learning, we compared the values of the (unconditional) entropy estimated from our spiking data within the premotor window T, with a reference Poisson spike train including a refractory period τ. When the bin size *dt* is equal to the window size T, the entropy of spike words is equivalent to the entropy of spike counts. One can then express its value exactly for a Poisson process with a refractory period from the exact analytical expression of the probability distribution of counts (Ferrari et al., 2018). On the other hand, in the limit where dt=1ms≪T, we use a previously-derived approximate analytical expression for the entropy of spike words of a refractory Poisson process (Nemenman et al., 2004). The refractory period we used is compatible with values obtained for the refractory period of RA neurons measured previously (Miller et al., 2017). We excluded from this plot all cases where we could not guarantee that only spikes from a single neuron were recorded. Indeed, having spikes from different neurons would artificially increase the variability and bias the entropy of spiking upwards.

## Figures and Tables

**Appendix 1-figure 1.**
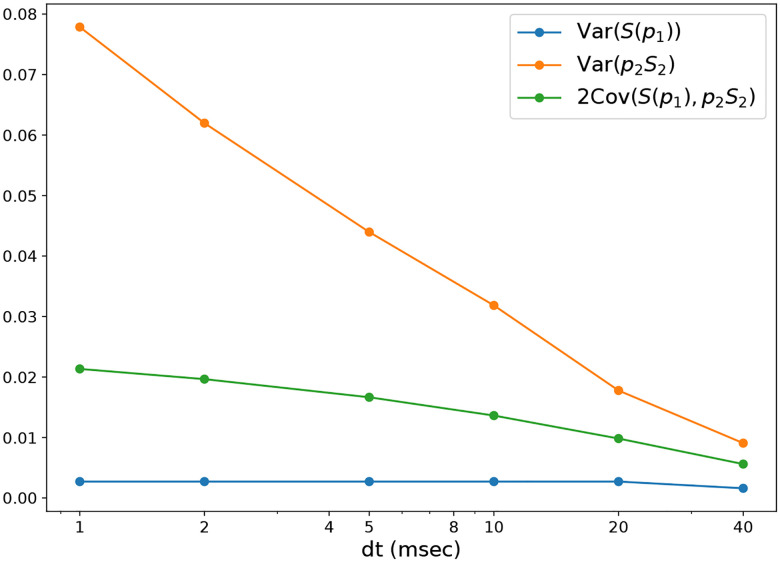
Contributions of the different terms in Equation 9 to the total variance of the partitioned unconditional entropy, as a function of binning size *dt*, for the spike words recorded during a typical syllable

**Appendix 2—figure 1.**
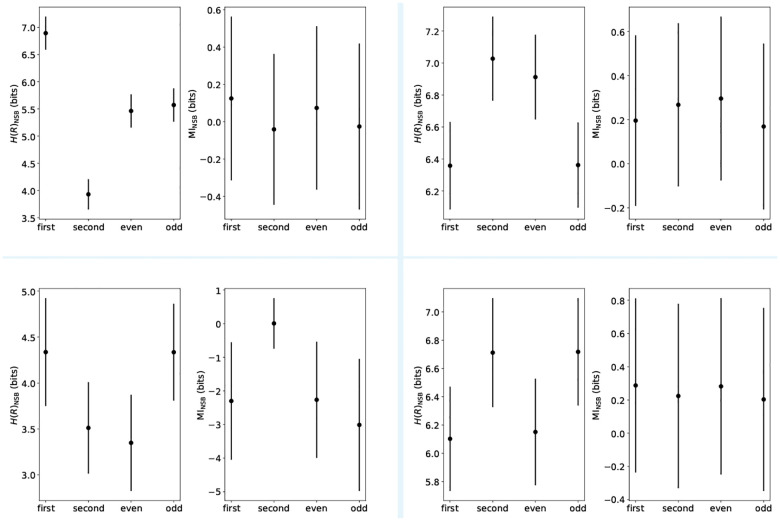
Results of tests for the presence of temporal correlations in our data. Data in each panel is calculated for a given syllable on a given day in a given bird. a) Bird 1 at age 68 dph, b) Bird 1 at 73 dph, c) Bird 2 at 81 dph and d) Bird 2 at 153 dph. All (unconditional) entropy estimates $H_{\text{NSB}}(R)$ and mutual information estimates ${\text{MI}}_{\text{NSB}}$ are given for the discretization *dt* = 1 ms. The different estimates in each panel were calculated from the first and second half of the total dataset, as well as for all even and odd trials in the dataset.

**Appendix 2—figure 2.**
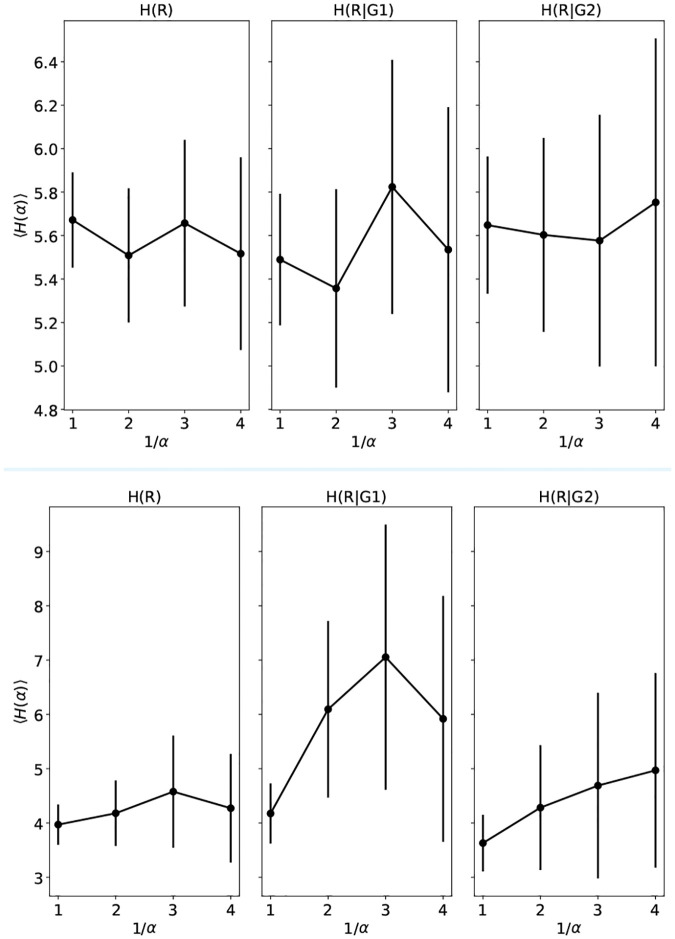
Finite data size scaling behavior of the unconditional entropy H(R) and the two entropies $H\left(R|G_{1}\right),H\left(R|G_{2}\right)$ which are conditional on the acoustic group $\left\{G_{1},G_{2}\right\}$. Estimates are plotted as a function of the inverse data fraction 1/α where αN is the number of randomly-selected trials that were used to compute the estimate. As 1/α→1, we can see that most entropy estimates are well within the error bars of the full estimate, hence confirming that our full dataset (i.e., α=1) are in the asymptotic regime of sufficient data size. a) Bird 1 at age 68 dph, b) Bird 2 at 81 dph. We used the same syllables as, respectively, a) and c) in Figure 1. All entropy estimates shown here were obtained for binning size *dt* = 1 ms. Convergence at larger *dt* is faster, and biases are even smaller.

## Appendix 1 Entropy partitioning

We start from the expression for the entropy of a disjoint mixture with $X_{1}$ and $X_{2}$ discrete random variables over disjoint alphabets. We set

(5)
$$X=\left\{\begin{array}{ll} X_{1} & \text{with}\;\text{probability}\;p_{1'}\\ X_{2} & \text{with}\;\text{probability}\;p_{2}=1-p_{1}. \end{array}\right.$$

The alphabet for variable $X_{1}$ comprises here only a single element, the most common word, with corresponding frequency of occurrence $p_{1}$. The alphabet for variable $X_{2}$ represents the set of all other words, with the overall frequency of occurrence $p_{2}$. We introduce the indicator function θ(X) such that

(6)
$$\theta (X)=\left\{\begin{array}{ll} 1 & \text{when}\;X=X_{1}\\ 2 & \text{when}\;X=X_{2} \end{array}\right.$$

Starting from the chain rule of conditional entropy (Cover and Thomas, 2012), we write

(7)
$$H\left(X,\theta \right)=H\left(\theta \right)+H\left(X|\theta \right)\;=H\left(\theta \right)+p\left(\theta =1\right)H\left(X|\theta =1\right)+p\left(\theta =2\right)H\left(X|\theta =2\right)\;=H\left(p_{1}\right)+p_{1}H\left(X_{1}\right)+\left(1-p_{1}\right)H\left(X_{2}\right)$$

with $H\left(p_{1}\right)=-p_{1}\text{log}p_{1}-\left(1-p_{1}\right)\text{log}\left(1-p_{1}\right)$ the “entropy of choice” between $X_{1}$ and $X_{2}$. As the alphabet of $X_{1}$ contains a single element, namely the most common word in the distribution, we get $H\left(X_{1}\right)=0$ and the formula for the partitioned entropy becomes

(8)
$$H=H\left(p_{1}\right)+p_{2}H_{2}.$$

We can then express the variance of the partitioned entropy as:

(9)
$$\text{Var}(H)=\text{Var}\left(H\left(p_{1}\right)\right)+\text{Var}\left(p_{2}H_{2}\right)+2\text{Cov}\left(H\left(p_{1}\right),p_{2}H_{2}\right).$$

To estimate the variance of the entropy of choice $H\left(p_{1}\right)$ we use the “delta method” (Hosmer Jr et al., 2008), which states that $\text{Var}[f(x)]\approx \text{Var}(x){\left[f^{'}(\text{E}(x))\right]}^{2}$. This gives

(10)
$$\text{Var}\left(H\left(p_{1}\right)\right)=\text{Var}\left(p_{1}\right){\left[{\text{log}}_{2}\left(\frac{1-\text{E}\left(p_{1}\right)}{\text{E}\left(p_{1}\right)}\right)\right]}^{2}.$$

We then use the fact that $p_{2}$ and $H_{2}$ are independent variables to write

(11)
$$\text{Var}\left(p_{2}H_{2}\right)={\left[E\left(p_{2}\right)\right]}^{2}\text{Var}\left(H_{2}\right)+{\left[E\left(H_{2}\right)\right]}^{2}\text{Var}\left(p_{2}\right)+\text{Var}\left(p_{2}\right)\text{Var}\left(H_{2}\right).$$

The variance of ${\text{H}}_{2}$ is obtained from the variance of the posterior distribution returned by the NSB estimator. The frequency of occurence $p_{2}$ for the set of all words that are not the most common word can be written as $p_{2}=n_{2}/N$, where $n_{2}$ is the number of times any word that is not the most common word appeared out of N trials. We then use the assumption that $n_{2}$ follows the binomial distribution with variance $Np_{2}\left(1-p_{2}\right)$ to get $\text{Var}\left(p_{2}\right)=p_{2}(1-\left.p_{2}\right)/N=\text{Var}\left(p_{1}\right)$. Finally, the last term in Equation 9 can be expressed as

(12)
$$2\text{Cov}\left(H\left(p_{1}\right),p_{2}H_{2}\right)=2E\left\{\left[H\left(p_{1}\right)-E\left(H\left(p_{1}\right)\right)\right]\left[p_{2}H_{2}-E\left(p_{2}H_{2}\right)\right]\right\},\;=2E\left[H\left(p_{1}\right)p_{2}H_{2}\right]-2E\left[H\left(p_{1}\right)\right]E\left(p_{2}H_{2}\right),\;=2E\left[H\left(p_{1}\right)p_{2}\right]E\left[H_{2}\right]-2E\left[H\left(p_{1}\right)\right]E\left(p_{2}\right)E\left(H_{2}\right),$$

where we used the fact that $p_{2}$ and $H_{2}$ are independent variables. Estimating the expectations $\text{E}\left[H\left(p_{1}\right)p_{2}\right]$ and $\text{E}\left[H\left(p_{1}\right)\right]$ up to the second moment in $p_{2}$, one obtains:

(13)
$$2\text{Cov}\left(H\left(p_{1}\right),p_{2}H_{2}\right)\approx \left[2{\text{log}}_{2}\left(\frac{1-\text{E}\left(p_{2}\right)}{\text{E}\left(p_{2}\right)}\right)-\frac{1}{{\text{log}}_{2}\left(1-\text{E}\left(p_{2}\right)\right)}+\frac{1}{\left(1-\text{E}\left(p_{2}\right)\right)\text{log}2}\right]\text{E}\left(H_{2}\right)\text{Var}\left(p_{2}\right).$$

Equation 9, together with Equation 10, Equation 11 and Equation 13 fully specify the error made on the estimate of the partioned entropy. The relative contributions from each term in Equation 9 are plotted in Figure 1.

## Appendix 2 Entropy bias analysis

### Time correlations

As stated in Entropy and mutual information estimation, we searched for significant temporal correlations in our data that could bias our entropy estimates. For this, we compared the difference in entropy estimates between the first and second half of all trials with the difference in entropy estimates between even and odd trials, see Figure 1.

As the difference in mean entropy between the first half and second half of our data was very similar to the difference in entropy between the two sets of alternating trials, we can assume that any temporal correlations in our data are very low and unlikely to affect our mutual information estimates.

### Finite data bias

We estimated the entropy S(α) for αN number of trials, with α<1 and N the total number of trials recorded, by randomly selecting trials and averaging over 10 realizations of this subsampling to yield the average entropy estimate ⟨S(α)⟩ for each data fraction α. Results of the data fraction analysis on the unconditional and conditional entropy estimates can be found in Figure 2.

As all entropy estimates for different data fractions agreed within error bars, we could confirm the absence of an empirical sample-size-dependent bias.
