# Millisecond-scale motor coding precedes sensorimotor learning in songbirds

**DOI:** 10.1101/2024.09.27.615500

**Published:** 2024-12-22

**Authors:** Leila May M. Pascual, Aanya Vusirikala, Ilya M. Nemenman, Samuel J. Sober, Michael Pasek

**Affiliations:** 1 Neuroscience Graduate Program, Emory University, Atlanta, United States;; 2 Department of Physics, Emory University, Atlanta, United States;; 3 Initiative in Theory and Modeling of Living Systems, Emory University, Atlanta, United States;; 4 Department of Biology, Emory University, Atlanta, United States

## Abstract

A key goal of the nervous system in young animals is to learn motor skills. Songbirds learn to sing as juveniles, providing a unique opportunity to identify the neural correlates of skill acquisition. Prior studies have shown that spike rate variability in vocal motor cortex decreases substantially during song acquisition, suggesting a transition from rate-based neural control to the millisecond-precise motor codes known to underlie adult vocal performance. By distinguishing how the ensemble of spike patterns fired by cortical neurons (the “neural vocabulary”) and the relationship between spike patterns and song acoustics (the “neural code”) change during song acquisition, we quantified how vocal control changes across learning in juvenile Bengalese finches. We found that despite the expected drop in rate variability (a learning-related change in spike vocabulary), the precision of the neural code in the youngest singers is the same as in adults, with 1–2 ms variations in spike timing transduced into quantifiably different behaviors. In contrast, fluctuations in firing rates on longer timescales fail to affect the motor output in both juvenile and adult animals. The consistent presence of millisecond-scale motor coding during changing levels of spike rate and behavioral variability suggests that learning-related changes in cortical activity reflect the brain’s changing its spiking vocabulary to better match the underlying motor code, rather than a change in the precision of the code itself.

## Introduction

Motor skills are acquired through repeated practice until they are performed at consistently high levels. During this process of motor refinement, the brain integrates sensory feedback and relays signals from motor regions of the brain to motor actuators in the periphery to shape behavior (Arber and Costa, 2018; Krakauer et al., 2019; Brainard and Doupe, 2002; Lemon, 2008). Studies that have examined how neural activity changes across learning have mostly quantified neural activity by measuring fluctuations in the spike rates of individual cortical neurons across hundreds of milliseconds during behavior. This approach, in studies primarily involving adult animals across many species, has consistently resulted in observations of spike rate variability changing across learning (Peters et al., 2014; Mandelblat-Cerf et al., 2009; Kargo and Nitz, 2004; Kojima et al., 2013; Cohen and Nicolelis, 2004).

Studies of neural changes during learning are especially lacking in developing animals, even though many survival-enabling skills are acquired before animals reach maturity (von Hofsten, 1991; Sullivan et al., 2008; Avitan et al., 2020; Adolph and Franchak, 2016; Mooney, 2009). In juvenile zebra finches, firing rate variability in motor cortical neurons has been shown to drop rapidly across vocal learning, although in adulthood neurons still showed heterogeneous levels of variability (Ölveczky et al., 2011). While these developmental reductions in variability might result from neurons reducing their range of spike rates (changes in spike rate vocabulary), they might also be affected by developmental changes in how particular spike patterns modulate behavior (changes in the motor code). This is especially crucial in songbirds, where changes in both cortical spiking activity (Mooney, 1992; Ölveczky et al., 2011; Vallentin et al., 2016; Miller et al., 2017; Yuan and Bottjer, 2019; Zemel et al., 2021) and the force-producing properties of vocal muscles (Adam and Elemans, 2020; Maxwell et al., 2021) change dramatically as animals mature.

**Figure 1.**
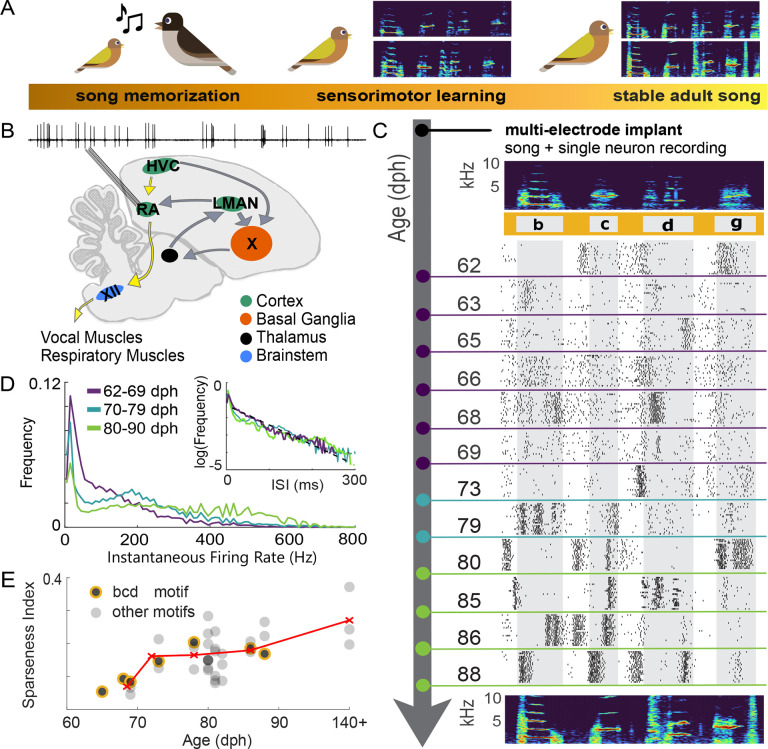
Recordings across song learning in juvenile Bengalese finches reveal changes in gross statistics of neural spiking. A) Song learning begins with early life exposure to a tutor song and continues through a period of sensorimotor practice. The two spectrograms depict a juvenile bird’s song during this period, which consists of distinct units of vocal gestures (syllables, identified with lowercase letters in panel C) that are highly variable from rendition to rendition. By adulthood, the two spectrograms depict the same syllables which are highly stereotyped and closely resembling the tutor’s song. B) Spiking activity from individual projection neurons in song motor nucleus RA is recorded across sensorimotor learning. Via the song motor pathway (SMP, yellow arrows), neurons in the motor nucleus RA send song motor commands to motor neurons in the brainstem nucleus nXIIts which directly innervate the vocal muscles. RA neurons receive input predominantly from HVC, a premotor nucleus upstream in the SMP, and from LMAN, the output nucleus of a thalamocortical basal ganglia loop which plays a necessary role in song learning (Mooney, 2009). C) The top panel is a spectrogram of a representative motif (a sequence of syllables ‘b’, ‘c’, ‘d’, and ‘g’) from a juvenile bird. The raster plots below show 20 representative spiking activities of isolated RA neurons during the song motif displayed in the spectrogram above. Each RA neuron (denoted by different colors) was recorded on a different day during song development. D) Across song learning, interspike interval distributions are reshaped from being approximately exponentially-distributed in early learning, to showing a bimodal ISI distribution typical of bursting activity in late learning. Instantaneous firing rates (1/ISI) during song, with a bimodal distribution clearly visible in late learning (days are grouped in purple, blue, and green). In earlier days, the ISI distribution closely follows the black dashed line, which corresponds to an exponentially-distributed ISI, equivalent to a Poisson model of spiking (inset). E) Sparseness index (SI) (Lehky et al., 2005; Ölveczky et al., 2011) of spiking across song learning. Black circles represent the average SI of an RA neuron recorded across iterations of a motif. Circles outlined in yellow represent SI of neurons recorded during the “bcd” motif shown in (C). Red ‘x’ markers represent the average for different age groups.

We sought to answer how the neural motor vocabulary and code evolve across developmental learning in the juvenile Bengalese finch, a species of songbird that learns its song through a period of sensorimotor learning prior to reaching sexual maturity at ~ 100 days post-hatch (dph, Figure 1A) (Fujii et al., 2021; Imai et al., 2016; Sasahara et al., 2015). Across song learning, juvenile Bengalese finches develop new vocal gestures called “syllables,” which are acoustically refined until being “crystallized” into stereotyped sequences by adulthood (Tchernichovski et al., 2001; Okanoya, 2004; Mooney, 2022). Our previous examination of spiking variations in adult Bengalese finches revealed that the information about rendition-to-rendition variations in syllable acoustics is present in precise (1–2 ms) variations in spike timing but absent in the firing rate (as measured by spike counts during a 40 ms window) (Tang et al., 2014; Hernández et al., 2022).

To understand the origins of this millisecond-precise motor code in adult songbirds, we recorded from neurons in vocal motor cortex – robust nucleus of the arcopallium (RA) – and examined the activity patterns preceding individual syllables at different developmental time points in juvenile Bengalese finches. Our analysis first shows that RA neurons in the juveniles display significant reductions in rate variability and increases in firing sparseness across the sensorimotor learning period, consistent with previous findings in zebra finches (Ölveczky et al., 2011). We then quantified how both the spiking vocabulary and the spiking code change across song acquisition by estimating, respectively, the entropy of each neuron’s repertoire of spiking patterns and the mutual information between spiking patterns and motor behavior (Nemenman, 2011; Tang et al., 2014).

We found that despite a dramatic developmental reduction in spike rate entropy (the size of the “vocabulary” of spike rates), the entropy (vocabulary size) of neurons’ precise timing patterns was nearly unchanged across vocal learning. Moreover, analysis of mutual information between syllable acoustics and RA spike trains at different temporal resolutions reveal a consistently precise motor code with millisecond-level structure in both juvenile and adult birds. These findings suggest that throughout sensorimotor learning, vocal production is driven by precise variations in spike timing, and that the maturation of the vocal code includes the gradual abandonment of ineffective timescales of neural variability (rate variation) while preserving the component of youthful variation (precisely-timed spike patterns) that is most effective at shaping behavior. Vocal skill learning might therefore be understood as the process of aligning the neural vocabulary with the neural code.

## Results

We recorded spiking activity from individual RA neurons in three male Bengalese finches together with song acoustics across the sensorimotor learning period (62–88 dph, see Subjects for age selection) and into adulthood (120–140 dph) (Figure 1 A). We collected data from 23 RA neurons across the three birds. We combined these data with a dataset from four adult (> 140 dph) Bengalese finches described in a previous study (Tang et al., 2014). We identified recorded neurons as putative projection neurons or interneurons based on their characteristic spike wave forms and spontaneous versus song spiking activity (see Electrophysiological recordings for details). In this dataset, 95% of isolated neurons were classified as putative projection neurons, consistent with our observations from previous recordings in adult birds (Tang et al., 2014; Sober et al., 2008). We therefore report data only from projection neurons since we did not record from enough interneurons to reliably quantify developmental changes in their activity.

### Emergence of bursting activity during song acquisition in Bengalese finches

Studies in adult zebra finches and Bengalese finches have shown that RA spiking activity is spontaneously tonic but strongly phasic during well-learned adult song, with RA spiking characterized by temporally precise, syllable-locked bursts (Yu and Margoliash, 1996; Chi and Margoliash, 2001; Leonardo and Fee, 2005; Sober et al., 2008; Wohlgemuth et al., 2010). While studies of RA spiking statistics in juvenile zebra finches (Ölveczky et al., 2011) suggest dramatic changes in the representation of vocal behavior across song acquisition, it is unknown whether or how the millisecond-precise spiking code described in adult Bengalese finches (Tang et al., 2014) emerges during song acquisition. Therefore, we recorded individual RA neurons from juvenile Bengalese finches to characterize gross changes in the statistics of firing activity across song learning (Figure 1 A,B,C,D). We found that late in the learning period (70–79 and 80–88 dph, blue and green traces in Figure 1 D) instantaneous firing rates (defined as the inverse of the interspike interval) show a bimodal distribution corresponding to high rates during bursts and low rates during inter-burst intervals. Early in the learning period, RA neurons produce a roughly exponential distribution of interspike intervals (ISIs) (62–69 dph, purple trace in Figure 1 D, inset).

To further characterize the reshaping of RA activity across song learning in Bengalese finches, we computed the sparseness index of firing patterns (Lehky et al., 2005; Ölveczky et al., 2011), defined as 1 when the spiking is restricted to a single time bin during a song motif and 0 if spikes are evenly distributed across the motif (Figure 1 E). We selected one to three motifs from each bird’s song, with each motif representing a different sequence comprised of three song syllables. We found that the sparseness index increased during development from 0.069 ± 0.017 (n=6) at 62–69 dph to 0.27 ± 0.09 in adult birds (140+ dph; n=3). These results show that, as previously observed in zebra finches, during song learning juvenile Bengalese finches exhibit a transition in activity from exponentially-distributed ISIs in RA neurons – typical of a Poisson spiking model – with a low sparsity in the early stages of learning to a bimodal firing rate distribution and sparse activity in later stages, as RA activity becomes dominated by brief, high-frequency bursts separated by epochs of silence (Yu and Margoliash, 1996; Leonardo and Fee, 2005; Sober et al., 2008).

### Spike rate variability and acoustic variability decrease together during learning

**Figure 2.**
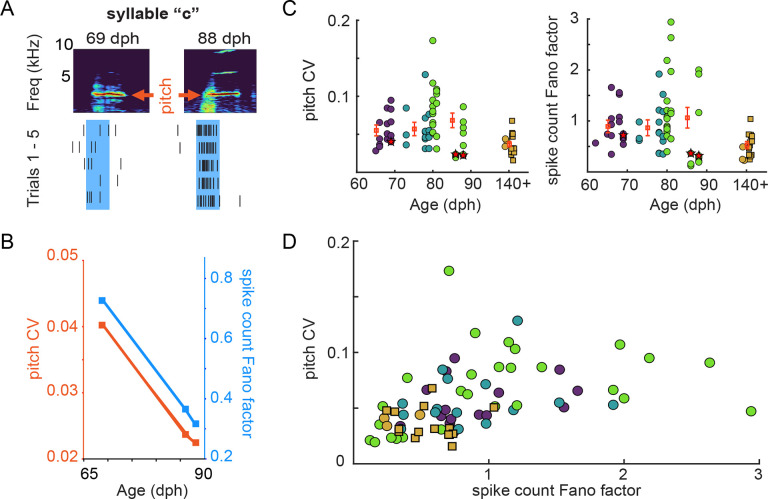
Variability of syllable acoustics and spike count measures of activity change drastically during song learning. A) Representative spectrograms of syllable ‘c’ during early learning (69 dph) and late learning (88 dph) reveal changes in acoustic structure, which we quantify across learning (e. g., as changes in the fundamental frequency, which we refer to here as “pitch”). Similarly, changes in RA spiking activity during the 40 ms premotor window (shaded area) are quantified across learning. B) The coefficient of variation of the pitch distribution of syllable ‘c’ (red symbols) decreases between 69 dph (CV = 0.04) and 88 dph (CV = 0.022). Spike count variability (Fano factor, blue symbols), concomitantly decreases during the same period (69 dph: Fano Factor = 0.72; 88 dph: Fano factor = 0.32). C) Pitch CV and spike count Fano factor for all analyzed syllables are depicted by individual circles. Values for the previously reported data (Sober et al., 2008; Tang et al., 2014) are shown as square symbols. The error bars show the mean ± SEM for each age group. Syllable ‘c’ at 69 dph, 86 dph, and 88 dph is depicted with stars. D) Variability in pitch correlates positively with variability in spike counts. Syllables from more mature birds (140+ dph) are predominantly in the lower left quadrant, indicating lower pitch and spike count variability compared with syllables from younger animals (circle and square symbols are color-coded for age as in C).

The observed global restructuring of spiking activity across learning warrants further exploration of the corresponding changes in both the vocabulary of motor commands (that is, which spike patterns are used by RA neurons) and the neural code (how these patterns are transduced into vocal acoustics). We therefore examined how spiking during individual vocal gestures (“syllables”) changes as song is learned, beginning by characterizing the variability of individual neurons’ spiking rate – namely, the total spike count in the “premotor window”, the epochs of RA activity that shape the acoustics of each syllable rendition. We define the premotor window as the 40 ms before the time at which the acoustic properties of each syllable were measured (Figure 2 A) as in our prior studies (Sober et al., 2008)). (Figure 2 A). To examine how rendition-by-rendition acoustic variability changed with the variability in RA spike rate vocabulary, we computed the coefficient of variation (CV) of the fundamental frequency (which we refer to as ‘pitch’) and the Fano factor of the spike count distribution during the premotor window for individual song syllables. A representative syllable “c” demonstrates the overall trajectory in acoustic and spike count variability between early learning and adult song: the pitch CV and the spike count Fano factor concomitantly decrease between early (69 dph) and late (86 and 88 dph) sensorimotor learning (Figure 2 B). Across all syllables and ages, the pitch distributions of individual syllables from juvenile birds (62–90 dph) showed wide ranging levels of variability with incidences of syllables with high variability values (CV > 0.1), while pitch variability was significantly smaller in adult birds (Figure 2 C), consistent with ranges previously shown in adult Bengalese (Tumer and Brainard, 2007; Sober et al., 2008; Sakata et al., 2008) and zebra finches (Kao and Brainard, 2006). We observed a similar trend in spike count distributions, which also showed wide ranging levels of variability from juvenile birds and significantly smaller variability in adult birds (Figure 2 C). Importantly, despite the heterogeneity in pitch and spike count variability levels during learning, we found that variability in pitch of individual syllables positively correlated with spike count variability (Figure 2 D).

The presence of syllables with high trial-to-trial variability in spike counts and acoustic features early in the sensorimotor learning period raises the possibility that trial-to-trial variations in acoustics are encoded by spike counts (Shadlen and Newsome, 1998; Brette, 2015). However, this analysis cannot distinguish if the overall decrease in trial-to-trial spike count variability and the associated acoustic variability during sensorimotor learning reflects an increase in the precision of the motor code. Alternatively, this decrease might be caused by a reduction in the vocabulary of spike counts at a fixed precision of the motor code. To distinguish these possibilities, we developed methods to separately quantify changes in the vocabulary of motor commands and the motor code itself, in both cases examining RA activity across the range of timescales from 1 msec to the total spike count in the 40 msec premotor window.

### Variability in millisecond-scale activity patterns remains constant across learning

**Figure 3.**
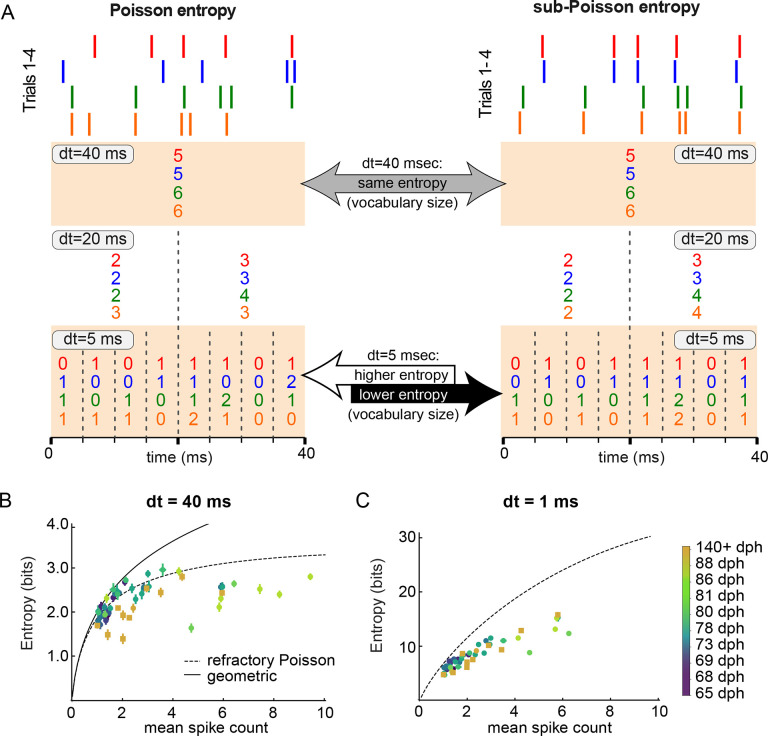
Entropy of RA activity at high temporal resolution suggests a non-Poisson neural spiking with statistical properties similar across days. A) Schematic for the representation of a 40 ms spike train discretized at different temporal resolutions *dt*. Each spike train represents a different trial of the activity of a given RA neuron during the 40 ms premotor window (see Text). Each number in the representation corresponds to the number of spikes in a specific bin of size *dt*. Left panel: four different spike trains from a refractory Poisson process. Observe that different spike trains can have the same representation at dt=40ms but different representations at higher temporal resolutions, e.g., dt=5ms. Intuitively, having different representations for different trials leads to a larger vocabulary at this resolution, and hence to a high value of entropy. In contrast, having the same representation across different trials leads to a smaller vocabulary and a lower value of entropy. Right panel: A neuron with reproducible spike patterns would have the same entropy as the refractory Poisson process at dt=40ms, but a lower entropy at higher temporal resolution. B) and C) Entropy of RA spike trains at different temporal resolutions as a function of the mean spike count during the premotor window. Entropy was calculated using the NSB method (see Entropy and mutual information estimation) at discretization of 40 ms for (B) and 1 ms for (C). Note that dt=40ms is equivalent to analyzing the overall spike count in the entire premotor window. Each neuron/day of recording is represented by a different color, from dark blue (age of 65 dph) to yellow (age > 140 dph), see color bar. We plot for comparison the entropy curve for a refractory Poisson process with the refractory period of τ=1ms, and the entropy of the geometric distribution of spike counts (the latter for dt=40ms only). Error bars represent one standard deviation of the entropy estimate, which are smaller than the symbol size in many cases.

Although we observed an overall decrease in the firing rate variability of RA neurons during the sensorimotor learning period, fully characterizing changes in the vocabulary of motor commands requires us to quantify the variability present at different timescales of RA activity. A suitable measure of variability is the entropy of the ensemble of spike trains – which can be discretized at different temporal resolutions – corresponding to the different renditions of a given motor output (Strong et al., 1998; Rieke et al., 1999) (see Entropy and mutual information estimation for the definition of entropy and details on its estimation). The resulting discretized activity for each rendition can be thought of as a spike “word” formed by a sequence of symbols, where each symbol represents the number of observed spikes during each time bin of size *dt*. This discretization is illustrated in Figure 3 A, which shows how spike times (Figure 3 A, top) can be represented as words at different timescales (Figure 3 A, bottom). We therefore represented the ensemble of spike words produced for each recorded neuron during each song syllable.

As schematized in Figure 3 A, two spike patterns that differ in their precise spike timing may be identical when discretized at a longer timescale. Figure 3 A shows synthetic spike trains with Poisson (left) and sub-Poisson (right) spiking variability, spike patterns in trial #1 (red) and trial #2 (blue) are identical when discretized at dt=40ms but different when discretized at dt=5ms. Thus, in Figure 3 B–C, we plot the entropy of spike words for individual syllables recorded at different developmental time points analyzed at two different timescales, dt=40ms (Figure 3 B) and dt=1ms (Figure 3 C). In both cases, we analyze the entropy as a function of the mean spike count during the premotor window, and we compare the entropy of the recorded vocabulary with the entropy of a refractory Poisson process with a refractory period τ=1ms, as well with the entropy of the geometric distribution (see Quantification of neural variability for details). When spike patterns are analyzed at the scale of the whole premotor window (dt=40ms, i.e., spike counts in the whole premotor window), we observe that the entropy of spike words in both adults (> 120 dph) and juveniles (65–88 dph) follows relatively closely the entropy of a refractory Poisson process as the mean spike count increases (Figure 3 B). Additionally, we found that vocabularies in adults (> 140 dph) tend to have lower entropy values than in juveniles at similar mean spike count (yellow symbols, Figure 3 B), confirming our findings that juvenile syllables show a higher degree of variability in trial-to-trial spike count values compared to adults. Although we observe some juvenile cases lying above the refractory Poisson entropy values, no adult syllable was found to show a similar high-entropy, “super-Poisson” behavior. Note that all entropy values were below the entropy of the geometric distribution, which defines the maximum entropy distribution at a particular mean spike count (Rieke et al., 1999).

To investigate spiking variability across both timescales and the process of sensorimotor learning, in Figure 3 C we plot the entropy of RA spike patterns discretized at a smaller resolution of dt=1ms, with a total word length set to 40 ms (the length of the premotor window) as above. In contrast to the developmental reduction in entropy at dt=40ms shown in Figure 3 B, the entropy (vocabulary size) of millisecond-precise spike patterns at dt=1ms was constant across the period of sensorimotor learning (Figure 3 C). Note that entropy values at dt=1ms (Figure 3 C) are larger than those at dt=40ms (Figure 3 B), as expected given the larger number of possible spike words at smaller timescales. Note also that all entropy values at dt=1 are below the entropy of a refractory Poisson spike train, regardless of age.

### Millisecond-scale motor coding of vocal acoustics throughout song learning

The contrast between the developmental reduction in the variability (vocabulary size) of spike patterns at dt=40ms with the constant level of variability of spike patterns at dt=1ms raises the question of which timescale of variability controls behavior (i.e., the timescale of the motor code) across learning. To answer this question, we estimated the mutual information between the activity patterns at different temporal resolutions and various parameters characterizing the vocal output. This parallels the analysis of Tang et al. (2014), but now we also estimate the mutual information as a function of the bird’s age. For three acoustic parameters – pitch, amplitude, and spectral entropy – we assigned every rendition of each song syllable to group 1 (or 2) if the syllable rendition was lower (or higher) than the median value of the acoustic parameter for all iterations of this syllable (see Methods).

**Figure 4.**
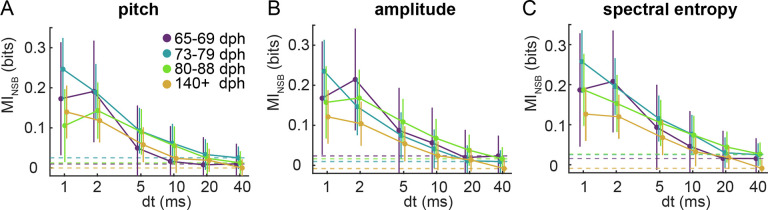
A precise timing code for vocal behavior control is observed in both juvenile and adult birds. Mutual information between the spike words and acoustic features at different temporal resolutions *dt*, as estimated by the NSB estimator (see Entropy and mutual information estimation). The three panels display values of mutual information between the spike train and three acoustic features: a) pitch, b) amplitude, and c) spectral entropy. Error bars represent one standard deviation (SD) of the information estimate, evaluated as in Entropy and mutual information estimation. We find that, in all three cases, and across all age categories, from early juvenile (65–69 dph, and 73–79 dph), to late juvenile (80–88 dph), and to adult, the mutual information increases as *dt* decreases, at least to about 2 ms. Additionally, the mutual information at the spike count scale, dt=40ms, is not statistically different from zero, indicating that variations in the spike rate do not have a measurable effect on variations in the studied acoustic features for each individual syllable. Adult neural and acoustic data (140+ dph) are from Tang et al. (2014), which we reanalyzed. Note that we consider two acoustic groups for each acoustic feature, thus the maximum possible shared information is one bit.

In Figure 4, we plot the mutual information estimates averaged over different syllables and different days in four different age categories (65–69 dph, 73–79 dph, 80–88 dph and > 140 dph), see Entropy and mutual information estimation for details. We found that the mutual information between the neural activity patterns syllable acoustics strongly depends on the resolution at which the neural pattern is analyzed, as was found previously in adult birds (Tang et al., 2014). Moreover, we found that variations in the pattern of activity of single RA neurons on timescales greater than 10 ms are not predictive of trial-to-trial variations of a syllable in any of the acoustic parameters examined, with mutual information values close to 0 bits. Crucially, we also found that the amount of information at the 1–2 ms timescale is similar for all age categories (Figure 4). This result suggests that the encoding of fine behavioral variations by ~ 1 ms fluctuations of spike timing of RA activity precedes the late stages of the sensorimotor learning period. This finding stands in stark contrast with expectations that the behavioral variability seen in juveniles stems from an as yet imprecise transformation of the neural activity into the motor output.

In our analysis, mutual information between the spike train and the acoustic features is a small difference between two large entropies: that of the vocabulary and that of the vocabulary conditioned on a behavioral group. Thus, relatively small biases in estimation of each of these entropies may result in large mutual information biases. While we worked hard to remove such biases, (see Entropy and mutual information estimation), it is crucial to provide an independent assessment of the quality of the mutual information estimation used here. For this, we note that all entropy (and hence mutual information) estimation biases are sample-size dependent (Paninski, 2003), typically decreasing polynomially as the sample size increases. Thus, if the increase of the mutual information at high temporal resolution in Figure 4 were due to residual estimation bias, we would expect the values to be larger for smaller sample sizes. To verify this, we repeated the mutual information estimation for different fractions of our total dataset (Figure 4 - figure Supplement 1). We observe that our main finding from Figure 4 – namely that information about trial-to-trial variations of individual syllables is encoded at the 1–2 ms resolution throughout the sensorimotor learning period – starts to disappear when the dataset size decreases by 50%. This supports the assertion that the increase of mutual information at small *dt* is not due to the estimation bias, but is a bona fide feature of the system.

## Discussion

Acquiring a motor skill challenges the developing animal to create new neural and behavioral patterns. To improve performance, the nervous system may change, perhaps simultaneously, the patterns of spikes produced during song (the spiking vocabulary) as well as the mapping of the vocabulary to the behavior (the motor code). In this study, we recorded single neurons in songbird cortical area RA to investigate the origins of the millisecond precise motor code known to underlie adult song production in the Bengalese finch. We found that the developing song system already uses millisecond-scale variations in its spiking vocabulary to control vocal acoustics, suggesting that the acquisition of skilled vocal performance is accomplished primarily through transformation of the spiking vocabulary rather than changes in the timescale of the motor code.

We found that sensorimotor learning in the Bengalese finch is accompanied by the emergence of bursting by RA neurons (Figure 1 C–E), consistent with prior findings in another songbird species (Ölveczky et al., 2011). When quantifying spike rate (the total spike count during the premotor window of individual song syllables), we found an overall decrease in spike count variability across the period of learning. This decrease in rate variability during learning suggests a hypothesis: that learning depends on the refinement of spike count patterns, as suggested previously in both songbirds and other systems (Dhawale et al., 2017; Costa, 2011).

A central goal of our study was to test this hypothesis. Moreover, we sought to understand the origins of the millisecond-precise motor code that underlies adult vocal production (Tang et al., 2014; Hernández et al., 2022; Srivastava et al., 2017). We found that RA neurons use a millisecond-scale motor code even as early at early stages of the sensorimotor learning period (Figure 4), and that variations in spike count were uncorrelated with behavioral variations throughout the sensorimotor learning period. Note that due to the difficulty of reliably identifying particular syllables in the songs of Bengalese finches younger than 60 dph (see Methods), these findings necessarily exclude the very earliest part of vocal practice in Bengalese finches.

In addition to quantifying the motor code, we used entropy measures to quantify developmental changes in the size of the vocabulary of spiking patterns. We found that despite the reduction in RA’s spike count vocabulary size between juvenile and adult song, the vocabulary of 1-ms spike patterns is maintained at consistent levels of variability from learning to adulthood (Figure 3 C). Since variations in spike rate do not appear to influence vocal acoustics, developmental reductions in spike rate variability might reflect songbirds’ refinement of their spiking vocabulary to eliminate timescales of spiking variation that do not affect behavior.

Developmental changes in the physiological properties and synaptic connectivity of RA neurons likely contribute to the reshaping of spiking vocabulary. Early in development, the intrinsic properties and excitability of RA neurons change significantly, however these changes are largely completed by 50 dph (Garst-Orozco et al., 2014; Zemel et al., 2021), prior to the age at which individual syllables can be reliably discriminated (see Methods). After 50 dph, instantaneous firing frequency in response to electrical current injections and spike rate adaptation do not change, although spike threshold and waveform show significant differences (Zemel et al., 2021). Therefore, the RA neurons we recorded during learning likely maintain relatively consistent responsiveness to external input arriving from two brain areas (Figure 1 B): LMAN, which guides vocal learning and injects variability into RA via the anterior forebrain pathway (Ölveczky et al., 2005, 2011; Kao et al., 2005) and HVC, which relay song timing signals via the motor pathway (Hahnloser et al., 2002; Leonardo and Fee, 2005; Long and Fee, 2008).

Developmental changes in spiking activity in RA’s two major input nuclei may also play a role in reshaping spiking vocabulary during learning. During learning, LMAN → RA synapses do not change significantly in strength or number (Garst-Orozco et al., 2014). However, patterns of LMAN spiking change markedly, increasing in trial-by-trial reliability in song-aligned spiking as well as increasing the prevalence of high-frequency bursts thought to increase variability in both RA spiking and vocal acoustics (Ölveczky et al., 2005; Kao et al., 2008; Kojima et al., 2013). HVC → RA connections, on the other hand, are first strengthened and then pruned as song matures, and modeling studies show that the effect of these changes is the eventual reduction in trial-to-trial variability of RA spike rate (Garst-Orozco et al., 2014). Changes in the spiking patterns of HVC → RA projection neurons might be influenced by learning-dependent increase in the recruitment of inhibitory interneurons in HVC, which have been shown to provide short-latency feedback inhibition to HVC projection neurons by adulthood (Vallentin et al., 2016; Kosche et al., 2015; Tupikov and Jin, 2021; Mooney and Prather, 2005). These observations suggest the possible roles for both HVC and LMAN in driving vocal changes during learning through modulating RA’s spiking vocabulary.

In addition to extrinsic inputs into RA, learning-related sculpting of lateral connections within RA may also contribute to changes in RA spiking vocabularies. Prior work has shown that song learning is associated with selective pruning of local inhibitory circuitry within RA: fast-spiking interneurons that were initially randomly connected to projection neurons become pruned while reciprocal connections are preserved (Miller et al., 2017). Furthermore, pharmacological disruption of interneuron activity leads to large shifts in learned pitch and amplitude output, suggesting that RA interneurons contribute to learning-related changes in the spiking vocabulary of RA projection neurons (as described in Unit Inclusion Criteria, our dataset consists entirely of putative RA projection neurons).

Millisecond-precise patterning of RA spikes can only influence vocal acoustics in juvenile animals if muscle actuators in the vocal periphery can transduce small variations in spike times into differences in muscle force and acoustic output. Importantly, a prior study examining the activation dynamics of songbird vocal muscles has shown that superfast muscles exist in the songbird syrinx prior to sensorimotor learning (Adam and Elemans, 2020). This indicates that even early in vocal learning, syringeal force production can be influenced by millisecond-scale variations in spike timing patterns (Srivastava et al., 2017; Adam and Elemans, 2020). Although developmental changes in the force producing properties of vocal muscles might influence the motor code, developmental changes in muscle contraction speed and magnitude are complete by 50 dph (Adam and Elemans, 2020). In addition, developmental changes in the passive (non-muscular) sound production properties of the vocal organ could impact how RA spiking vocabularies shape acoustic output. However, in zebra finches, the passive sound-producing properties of the syrinx do not change across song development (Maxwell et al., 2021). Together, these studies suggest that the properties of the vocal apparatus and vocal muscles do not change significantly during sensorimotor learning; therefore changes in the vocabulary of central motor commands seem most likely to drive vocal changes during learning.

Crucially, our mutual information analysis (Figure 4) shows that the temporal precision of the neural code—but not necessarily the code itself—is invariant during learning. Given that neither the timescale of motor coding nor the vocabulary size in that timescale changes across learning, the drivers of learning-related vocal changes might therefore include changes in the content of RA neurons’ vocabularies of 1-ms spike patterns (i.e., the distribution of unique 1-ms spike patterns, or codewords). Detecting specific codewords, as in Hernández et al. (2022) and analyzing their behavioral consequences across learning is essential for tracking the evolution of the (millisecond-precise) code itself. This would require an ability to record spiking activity from the same neuron for days or weeks at a time. While this is still beyond reach in the songbird system, the existence of a precise code even early in learning, which we showed here, adds urgency to developing the necessary technology.

## Methods and Materials

### Subjects

Male Bengalese finches (*Lonchura striata var. Domestica*, songbird species of which only males learn to sing) were bred in our colony and housed with their parents until 60 days of age. After electrode implantation, birds were isolated and housed individually in sound-attenuating chambers with food and water provided *ad libitum*. All recordings are from undirected song (i.e., no female was present). Recordings from three juvenile birds (60–100 dph) were collected using a previously described experimental protocol (Sober et al., 2008). Although sensorimotor learning in Bengalese finches typically begin ~ 40 dph (Imai et al., 2016; Sasahara et al., 2015), we chose 60 dph as the earliest age for our analysis for two reasons. First, song syllables from days earlier than 60 dph are acoustically difficult to quantify and categorize into distinct syllable identities, constraining our ability to quantify within-syllable acoustic variation. Second, the surgical challenges of implanting younger Bengalese finches include their fragile, immature skull; attempting to secure a microdrive on their skulls at younger ages would compromise our ability to hold single neurons for longer periods as well as the sturdiness of the implant through the weeks-long chronic recordings. A subset of the recordings from three adult birds were collected from the same juvenile birds that had matured into adulthood. The remaining subset of recordings from four adult birds (>140 dph) were previously collected as part of a separate analysis (Sober et al., 2008). Procedures were performed in accordance with established animal care protocols approved by the Emory University Institutional Animal Care and Use Committee.

### Song Acoustic Analysis

#### Song Detection

A microphone placed inside the housing chamber chronically recorded song simultaneously with neural recordings. To quantify acoustics of song syllables, we first detected the presence and identity of song syllables from the audio recordings. Syllable onset and offset times were determined on the basis of amplitude threshold crossings after smoothing the acoustic waveform with a square filter of width 2 ms. The error of our determination of syllable onset time is therefore on the order of milliseconds. This millisecond-scale uncertainty in syllable onset time cannot account for our results since millisecond-scale jitter would decrease, rather than increase, our information estimates at fine timescales.

#### Syllable Acoustic Quantification

The identities of song syllables were determined by visual examination of spectrogram renderings of song behavior. For each syllable identity, we determined a particular time (relative to syllable onsets) when the spectral features were well defined to quantify vocal acoustics. This time of quantification was taken to be fixed relative to the syllable onset, i.e., it does not vary between different iterations of a given syllable. We analyzed the acoustic power spectrum at the specified time during each iteration of a song syllable and quantified the fundamental frequency (which we refer to as “pitch”), amplitude, and spectral entropy. We chose to quantify these three acoustic features since they capture a large percentage of acoustic variability in Bengalese finch song and are the features that are refined during song learning (Sober et al., 2008).

### Electrophysiological recordings

#### Surgical Implantation of Microdrive

Birds were anesthetized (induction with 3% isoflurane, maintained with 1.5–2% isoflurane) and a lightweight 16-microelectrode bundle microdrive (Innovative Neurophysiology) was stereotactically positioned above RA nucleus in one hemisphere (2 implants over right RA, 1 over left RA) and secured to the skull with dental cement. After birds recovered from surgery and singing resumed (within 1–3 days), electrodes were advanced through RA using a miniaturized microdrive which recorded extracellular voltage traces during and between bouts of singing. RA recording sites were confirmed by the presence of characteristic changes in activity associated with the production of song and calls and by *post hoc* histological confirmation of electrode tracks passing through the RA nucleus.

#### RA Unit Isolation

To isolate the spiking activity from individual units, we used a previously-described spike sorting algorithm (Sober et al., 2008), which provided a scalar measurement of unit isolation to establish a quantitative-based inclusion criterion for our spike train analyses. Briefly, we determined a voltage threshold to detect both spike and noise waveforms from singing-related activity. We then performed principal components analysis (PCA) on these waveforms and used the first two principal components to project a 2-dimensional representation of the waveforms. We then assigned each waveform to a cluster by applying an automated nearest-neighbor clustering algorithm (kmeans.m in MATLAB, The MathWorks, MA) to the 2-dimensional data. For the majority of cases, two clusters were selected: a “spike” and a “noise” cluster.

We obtained a measure of a unit’s isolation by quantifying the extent of overlap between the spike and noise clusters. To do this, we fit a 2-D Gaussian to each cluster to estimate the mean and variance of each cluster. We then used the Gaussian fits to generate 10,000 synthetic points for each respective cluster. We re-applied the nearest-neighboring algorithm on the synthetic points and quantified the extent of cluster overlap by the frequency, with which synthetic points were miscategorized by the algorithm. We deemed that a frequency of less than 0.01 of cluster overlap (or “isolation error”) was a reasonable threshold for classifying the spike cluster as a single-unit.

#### Unit Inclusion Criteria

We examined the interspike interval (ISI) distributions of each successfully isolated single-unit. Only single units with < 1% of interspike intervals less than 1 millisecond were included in our analysis or RA activity from juvenile birds. Based on prior characterizations cell-type specific spike waveform shape and response properties (Sober et al., 2008), RA recordings were classified as putative excitatory projection neurons or interneurons, with >97% of neurons identified as putative projection neurons. Only RA projection neurons were included in the analysis for this study. In total, we collected 30 RA single-unit recordings. Recordings spanned 27 days across song learning for Bird 1, 5 days for Bird 2, and 3 days for Bird 3.

### Spiketrain analysis

#### Entropy and mutual information estimation

To quantify the amount of information on vocal acoustics that is conveyed by the activity of RA neurons at different timescales, we estimated the mutual information (Shannon, 1948) between discretized measures of neural activity within the premotor window and the “behavioral group” of syllables, based on acoustic features of iterations of individual song syllables or hand-labelling syllable identities across different syllables. We did this at different temporal resolutions of the neural activity, as is commonly done (Strong et al., 1998; Nemenman et al., 2004; Tang et al., 2014). As in previous analyses (Tang et al., 2014; Hernández et al., 2022), the premotor window was defined relative to the time of quantification for acoustic features and taken to be of length T=40ms.

Within the premotor window, we discretize the spiketrain into bins of duration *dt*, with 1≤dt≤40ms. We then digitize the spiketimes, i.e. set the value in each time bin to the number of spikes in that bin. Given the existence of a refractory period $\tau_{\text{ref}}$ in RA neurons of approximately 2 ms (Miller et al., 2017; Zemel et al., 2021), we do not expect more than one spike to be present per 1 ms bin, but larger *dt* can result in more than one spike per bin. Thus, the spike times in each premotor window are translated into a spike word containing T/dt digits, with each digit equal to the spike count observed within each time bin of size *dt*.

To analyze the properties of the neural code at different timescales, we perform our mutual information analysis at different time resolutions dt={1, 2,5, 10, 20, 40}ms (these values fit into the premotor window T=40ms integer number of times, and they are roughly uniformly spread when plotted on a log-scale axis).

Using these $N_{\text{trials}}$ spike words R of size T/dt, together with the $N_{\text{trials}}$ acoustic group indices G for each acoustic feature, one can then estimate the mutual information between the spike train and the motor output through a difference in two entropies (Tang et al., 2014)

(1) $$I_{T,\text{dt}}(R;G)=H_{T,\text{dt}}(R)-{\left\langle H_{T,\text{dt}}(R|G)\right\rangle }_{G},$$

where $\langle \ldots \rangle_{G}$ represents an average over the behavioral group G. The mutual information Equation 1 quantifies the reduction in uncertainty of a random variable R, as measured by the entropy H(R), due to the knowledge of another random variable G, as quantified by the conditional entropy H(R∣G) (Cover and Thomas, 2012).

Given a probability distribution p(R), the entropy H(R) is defined as

(2) $$H(R)=-\sum_{R}\;p(R){\text{log}}_{2}\;p(R).$$

Obtaining an accurate estimate of the entropy from finite datasets is a notoriously difficult problem (Paninski, 2003), as it requires an estimatiion of the probability distribution p(R) from a finite number of samples. In the asymptotic sampling regime, where each response R has been sampled multiple times, the “naive” or “maximum likelihood” estimate can be used, which replaces probabilities by empirically observed word frequencies $p(R)\approx f(R)=n_{R}/N$ (where $n_{R}$ is the number of times a specific response R was observed, and N is the total number of different responses) in the definition of the entropy Equation 2. Such an estimate of the entropy is biased, with the bias converging to zero as N→∞ (Paninski, 2003). The convergence is slow, ~|R|/N, where |R| is the cardinality of the variable, whose entropy is being analyzed. However, often one is interested in estimating the entropy for distributions over the space of possible responses with a very large cardinality. The cardinality of possible responses depends exponentially on the size of the time window T and the time binning resolution *dt*, and can, in principle, be very large, precluding the use of the naive estimator.

Indeed, we estimate the cardinality $|R{|}_{\text{est}}$ of the observed spike trains using the standard estimate for the number of “typical” sequences when drawing n independent and identically distributed discrete random variables (known as the “asymptotic equipartition property”) (Cover and Thomas, 2012):

(3) $$|R{|}_{\text{est}}=2^{nH_{\text{est}}\left(R\right)},$$

where n=T/dt is the number of bins used to discretize the spike trains, and we take the entropy estimate $H_{\text{est}}(R)$ to be the entropy of a Poisson distributed counts for the number of spikes in each bin with mean $\lambda =\left(\left\langle n_{\text{sp}}\right\rangle +3\sqrt{\left\langle n_{\text{sp}}^{2}\right\rangle -{\left\langle n_{\text{sp}}\right\rangle }^{2}}\right)\text{dt}/T$, where $n_{\text{sp}}$ is the total number of spikes observed during the premotor window T in each trial, and ⟨…⟩ represents an average over trials. At the finest discretization considered here, dt=1ms, and for a typical average spike count, we find the number of possible neural words is on the order of $|R{|}_{\text{est}}\sim 10^{10}$. As this cardinality is much higher than the typical number of trials per group (n≳200) that we were able to collect in our data, we face the task of giving a reliable estimate of entropy in the severely undersampled regime.

Because of this constraint, we use the Nemenman-Shafee-Bialek (NSB) entropy estimation technique (Nemenman et al., 2001; Nemenman, 2011; Hernández et al., 2023). This is a Bayesian approach that provides an estimate of the entropy, together with an error bar on the estimate that is the standard deviation of the posterior distribution. It has no systematic bias for short-tailed distributions (Hernández et al., 2023) – in contrast to the maximum-likelihood estimate – even in the strongly undersampled regime. The ability of the NSB approach to give an unbiased entropy estimate in the undersampled regime lies in its dependence on counting the number of “coincidences” in the dataset, i.e., the number of times each spike “word” is seen more than once over repeated trials (Nemenman, 2011). As a reliable entropy estimate can be provided by the NSB approach only if the number of coincidences in the data is significantly greater than one, we verified that this criterion is satisfied for the observed word distributions in all syllables and acoustic groups. We found that the number of coincidences in the data was high enough to yield a reliable estimate of entropy when the minimum number of trials for each individual syllable was greater than 200. Only these syllables were included in our analysis. Additionally, we restricted our analysis to syllables for which mean spike count values were greater than one, as this corresponds to our threshold for defining an RA unit as “active” (Sober et al., 2008). A total of 46 syllables across 3 birds and ages from 65 dph to 153 dph satisfied this criterion.

The NSB approach to estimating the entropy relies on having a fast (e.g., exponential) decay of the rank-ordered distribution of words as a function of the rank (Nemenman, 2011; Hernández et al., 2023) (see also Camaglia et al. (2024)). In particular, entropy estimates for rank-ordered distributions of words with long (e.g., power law) tails tend to underestimate the true entropy (Nemenman et al., 2004, 2008). To circumvent this problem, we make use of an exact relation for the additivity of entropy for a mixture of two disjoint partitions of data (i.e., containing no overlapping words), see Appendix 1 for a derivation of the formula for the additivity of entropy and the corresponding error bars on the total estimate. Thus, similarly to Nemenman et al. (2008); Tang et al. (2014), we partitioned trials into the most common word vs. all other words in cases (i.e., for a given syllable on a given day) where the most common spike word in the distribution appeared in more than 2% of the recorded trials. For juvenile birds, the most common word is in all cases the “silence” word with no spikes.

To find out if our entropy estimates are indeed free from any statistical bias given the limited dataset sizes we could reach in our experiments, we performed a series of tests, to verify that: firstly, temporal correlations across different trials are low and the different spike words can be assumed to be independent samples of the underlying word distribution, and secondly, that our dataset does not suffer from undersampling bias. We give a short description of these two tests below, following Tang et al. (2014), while the results of these tests can be found in Appendix 2.

The absence of significant temporal correlations in the spiking data for every syllable/neuron in the course of a single day was assessed by comparing the difference in entropy estimates between the first and second half of all trials, with the difference in entropy estimates between even and odd trials. The latter is taken as a reference, where any contribution from long time correlation effects should be cancelled out. As these two differences in entropy estimates were comparable, we ruled out the presence of significant temporal correlations over the course of each day.

To address the potential issue of finite sample bias in our data, we verified that individual conditional and unconditional entropy estimates showed little variations when computed from smaller data fractions than our complete dataset (Strong et al., 1998; Nemenman et al., 2008; Tang et al., 2014). More specifically, when estimating the entropy for a case with N number of trials recorded, we also estimated the entropy H(α) for αN, with α<1, randomly selected trials and averaged over 10 realizations of this subsampling to yield the subsampled-average entropy estimate ⟨H(α)⟩. By observing the finite data size scaling behavior of ⟨H(α)⟩ as a function of the inverse data fraction 1/α→1, we could confirm that a large fraction of our entropy estimates were in an asymptotic regime of sufficient data size.

To obtain the mutual information curves for each age range in Figure 4, we averaged the mutual information estimate Equation 1, first over all cases recorded in each day, and then over the different recorded days in each age range. The averaging in both cases was performed by “inversevariance weighting”, i.e., by weighing the entropy contribution of each case by the inverse of the posterior variance of its estimate, as in Tang et al. (2014). The error bar on the mutual information average for each age range in Figure 4 represents the standard deviation of the mean, and was similarly calculated by averaging the individual posterior variance of each estimate, first for all cases in each day, and then over the different recorded days in each age range. These were again weighed by the inverse of the posterior variance of the corresponding estimate. To further confirm the absence of undersampling bias in our data, we repeated the data fraction analysis described above on the final averaged mutual information estimates. We observe that our main finding from Figure 4 – namely that mutual information between spike timing and behavioral variation within a given syllable is higher at finer resolution, dt=1ms – is preserved when we subsample our dataset down to 50% of its full size, see Appendix 2 and figure Supplement 1.

#### Quantification of neural variability

A standard way of estimating the variability of spike trains is to calculate the Fano factor (Gerstner et al., 2014)

(4) $$F=\frac{\langle {\left(\Delta n_{\text{sp}}\right)}^{2}\rangle }{\langle n_{\text{sp}}\rangle },$$

which allows to compare the variability of spike trains with different mean firing rates $\left\langle n_{\text{sp}}\right\rangle /T$. If the distribution of spike counts is modeled as an homogeneous Poisson process, where random occurrences of spikes are independent and happen with a constant rate, the Fano factor is exactly one. This allows to gauge neural variability by measuring its distance to Poisson-like firing. However, the Fano factor gives an estimate of the variability only at the relatively coarse scale of the measured spike count during time window T. Additionally, RA neurons have been observed to display a refractory period (which forbids mutiple spikes from happening in close succession), a fact that is not taken into account by the simple Poisson process.

To quantify the variability in spiking activity at different scales, including millisecond timescale, and different time points during learning, we compared the values of the (unconditional) entropy estimated from our spiking data within the premotor window T, with a reference Poisson spike train including a refractory period τ. When the bin size *dt* is equal to the window size T, the entropy of spike words is equivalent to the entropy of spike counts. One can then express its value exactly for a Poisson process with a refractory period from the exact analytical expression of the probability distribution of counts (Ferrari et al., 2018). On the other hand, in the limit where dt=1ms≪T, we use a previously-derived approximate analytical expression for the entropy of spike words of a refractory Poisson process (Nemenman et al., 2004). The refractory period we used is compatible with values obtained for the refractory period of RA neurons measured previously (Miller et al., 2017). We excluded from this plot all cases where we could not guarantee that only spikes from a single neuron were recorded. Indeed, having spikes from different neurons would artificially increase the variability and bias the entropy of spiking upwards.

## Supplementary Material

1 **Figure 4—figure supplement 1.** Behavior of the mutual information as a function of the data set size supports the hypothesis of a temporally precise neural code across the sensorimotor period.

## Figures and Tables

**Appendix 1—figure 1.**
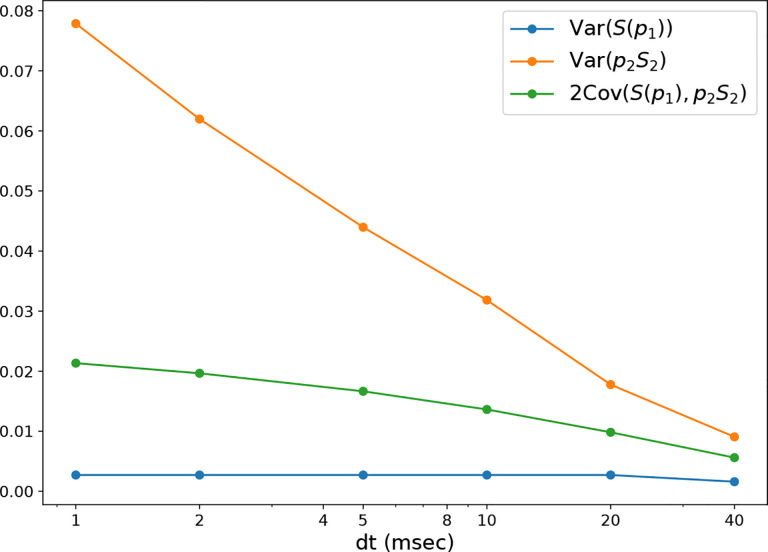
Contributions of the different terms in Equation 9 to the total variance of the partitioned unconditional entropy, as a function of binning size *dt*, for the spike words recorded during a typical syllable

**Appendix 2—figure 1.**
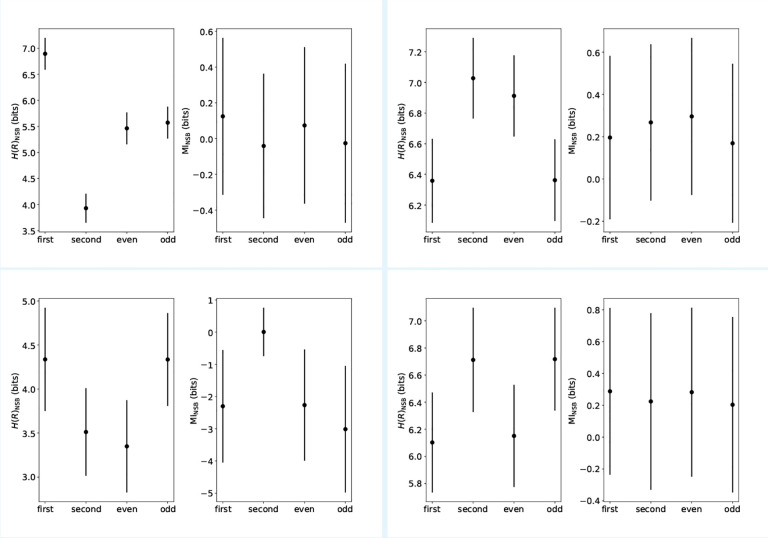
Results of tests for the presence of temporal correlations in our data. Data in each panel is calculated for a given syllable on a given day in a given bird. a) Bird 1 at age 68 dph, b) Bird 1 at 73 dph, c) Bird 2 at 81 dph and d) Bird 2 at 153 dph. All (unconditional) entropy estimates $H_{\text{NSB}}(R)$ and mutual information estimates MI_NSB_ are given for the discretization dt=1ms. The different estimates in each panel were calculated from the first and second half of the total dataset, as well as for all even and odd trials in the dataset.

**Appendix 2—figure 2.**
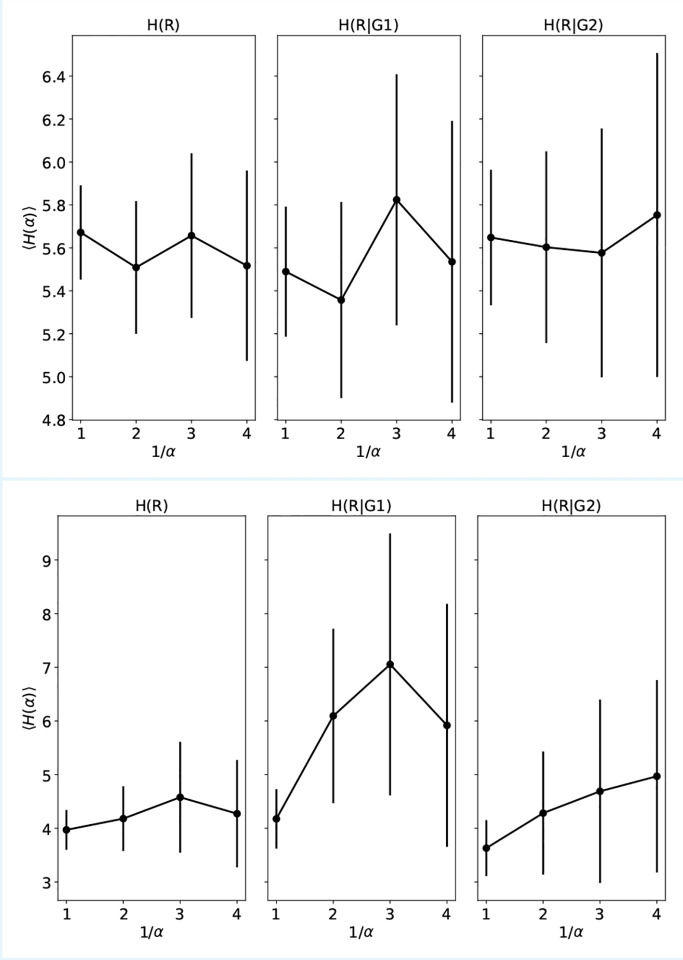
Finite data size scaling behavior of the unconditional entropy H(R) and the two entropies $H\left(R|G_{1}\right),H\left(R|G_{2}\right)$ which are conditional on the acoustic group $\left\{G_{1},G_{2}\right\}$. Estimates are plotted as a function of the inverse data fraction 1/α where αN is the number of randomly-selected trials that were used to compute the estimate. As 1/α→1, we can see that most entropy estimates are well within the error bars of the full estimate, hence confirming that our full dataset (i.e., α=1) are in the asymptotic regime of sufficient data size. a) Bird 1 at age 68 dph, b) Bird 2 at 81 dph. We used the same syllables as, respectively, a) and c) in Figure 1. All entropy estimates shown here were obtained for binning size dt=1ms. Convergence at larger *dt* is faster, and biases are even smaller.

## Appendix 1

### Entropy partitioning

We start from the expression for the entropy of a disjoint mixture with $X_{1}$ and $X_{2}$ discrete random variables over disjoint alphabets. We set

(5)
$$X=\left\{\begin{array}{ll} X_{1} & \text{with}\;\text{probability}\;p_{1},\\ X_{2} & \text{with}\;\text{probability}\;p_{2}=1-p_{1}. \end{array}\right.$$

The alphabet for variable $X_{1}$ comprises here only a single element, the most common word, with corresponding frequency of occurrence $p_{1}$. The alphabet for variable $X_{2}$ represents the set of all other words, with the overall frequency of occurrence $p_{2}$. We introduce the indicator function θ(X) such that

(6)
$$\theta (X)=\left\{\begin{array}{ll} 1 & \text{when}\;X=X_{1}\\ 2 & \text{when}\;X=X_{2} \end{array}\right.$$

Starting from the chain rule of conditional entropy (Cover and Thomas, 2012), we write

(7)
$$H\left(X,\theta \right)=H\left(\theta \right)+H\left(X|\theta \right)\;=H\left(\theta \right)+p\left(\theta =1\right)H\left(X|\theta =1\right)+p\left(\theta =2\right)H\left(X|\theta =2\right)\;=H\left(p_{1}\right)+p_{1}H\left(X_{1}\right)+\left(1-p_{1}\right)H\left(X_{2}\right)$$

with $H\left(p_{1}\right)=-p_{1}\;\text{log}\;p_{1}-\left(1-p_{1}\right)\;\text{log}\left(1-p_{1}\right)$ the “entropy of choice” between $X_{1}$ and $X_{2}$. As the alphabet of $X_{1}$ contains a single element, namely the most common word in the distribution, we get $H\left(X_{1}\right)=0$ and the formula for the partitioned entropy becomes

(8)
$$H=H\left(p_{1}\right)+p_{2}H_{2}.$$

We can then express the variance of the partitioned entropy as:

(9)
$$\text{Var}(H)=\text{Var}\left(H\left(p_{1}\right)\right)+\text{Var}\left(p_{2}H_{2}\right)+2\text{Cov}\left(H\left(p_{1}\right),p_{2}H_{2}\right).$$

To estimate the variance of the entropy of choice $H\left(p_{1}\right)$ we use the “delta method” (Hosmer Jr et al., 2008), which states that $\text{Var}[f(x)]\approx \text{Var}(x){\left[f^{'}(E(x))\right]}^{2}$. This gives

(10)
$$\text{Var}\left(H\left(p_{1}\right)\right)=\text{Var}\left(p_{1}\right){\left[{\text{log}}_{2}\;\left(\frac{1-E\left(p_{1}\right)}{E\left(p_{1}\right)}\right)\right]}^{2}.$$

We then use the fact that $p_{2}$ and $H_{2}$ are independent variables to write

(11)
$$\text{Var}\left(p_{2}H_{2}\right)={\left[E\left(p_{2}\right)\right]}^{2}\text{Var}\left(H_{2}\right)+{\left[E\left(H_{2}\right)\right]}^{2}\text{Var}\left(p_{2}\right)+\text{Var}\left(p_{2}\right)\text{Var}\left(H_{2}\right).$$

The variance of $H_{2}$ is obtained from the variance of the posterior distribution returned by the NSB estimator. The frequency of occurence $p_{2}$ for the set of all words that are not the most common word can be written as $p_{2}=n_{2}/N$, where $n_{2}$ is the number of times any word that is not the most common word appeared out of N trials. We then use the assumption that $n_{2}$ follows the binomial distribution with variance $Np_{2}\left(1-p_{2}\right)$ to get $\text{Var}\left(p_{2}\right)=p_{2}(1-\left.p_{2}\right)/N=\text{Var}\left(p_{1}\right)$. Finally, the last term in Equation 9 can be expressed as

(12)
$$2\text{Cov}\left(H\left(p_{1}\right),p_{2}H_{2}\right)=2E\left\{\left[H\left(p_{1}\right)-E\left(H\left(p_{1}\right)\right)\right]\left[p_{2}H_{2}-E\left(p_{2}H_{2}\right)\right]\right\},\;=2E\left[H\left(p_{1}\right)p_{2}H_{2}\right]-2E\left[H\left(p_{1}\right)\right]E\left(p_{2}H_{2}\right),\;=2E\left[H\left(p_{1}\right)p_{2}\right]E\left[H_{2}\right]-2E\left[H\left(p_{1}\right)\right]E\left(p_{2}\right)E\left(H_{2}\right),$$

where we used the fact that $p_{2}$ and $H_{2}$ are independent variables. Estimating the expectations $E\left[H\left(p_{1}\right)p_{2}\right]$ and $E\left[H\left(p_{1}\right)\right]$ up to the second moment in $p_{2}$, one obtains:

(13)
$$2\text{Cov}\left(H\left(p_{1}\right),p_{2}H_{2}\right)\approx \left[2\;{\text{log}}_{2}\left(\frac{1-E\left(p_{2}\right)}{E\left(p_{2}\right)}\right)-\frac{1}{{\text{log}}_{2}\left(1-E\left(p_{2}\right)\right)}+\frac{1}{\left(1-E\left(p_{2}\right)\right)\text{log}\;2}\right]E\left(H_{2}\right)\text{Var}\left(p_{2}\right).$$

Equation 9, together with Equation 10, Equation 11 and Equation 13 fully specify the error made on the estimate of the partioned entropy. The contributions from each term in Equation 9 are plotted in Figure 1.

## Appendix 2

### Entropy bias analysis

#### Time correlations

As stated in Entropy and mutual information estimation, we searched for significant temporal correlations in our data that could bias our entropy estimates. For this, we compared the difference in entropy estimates between the first and second half of all trials with the difference in entropy estimates between even and odd trials, see Figure 1.

As the difference in mean entropy between the first half and second half of our data was very similar to the difference in entropy between the two sets of alternating trials, we can assume that any temporal correlations in our data are very low and unlikely to affect our mutual information estimates.

#### Finite data bias

We estimated the entropy S(α) for αN number of trials, with α<1 and N the total number of trials recorded, by randomly selecting trials and averaging over 10 realizations of this subsampling to yield the average entropy estimate ⟨S(α)⟩ for each data fraction α. Results of the data fraction analysis on the unconditional and conditional entropy estimates can be found in Figure 2.

As all entropy estimates for different data fractions agreed within error bars, we could confirm the absence of an empirical sample-size-dependent bias.
