# Supplementary material for: Millisecond-scale motor coding precedes sensorimotor learning in songbirds
Source: bioRxiv. 2024 Dec 22:2024.09.27.615500. Originally published 2024 Sep 27. Preprint. [Version 2] doi: 10.1101/2024.09.27.615500 (PMC11463345; doi:10.1101/2024.09.27.615500)
Supplement: 1 — Figure 4—figure supplement 1. Behavior of the mutual information as a function of the data set size supports the hypothesis of a temporally precise neural code across the sensorimotor period. [file NIHPP2024.09.27.615500v2-supplement-1.pdf]

bioRxiv preprint doi: https://doi.org/10.1101/2024.09.27.615500; this version posted December 22, 2024. The copyright holder for this preprint (which was not certified by peer review) is the author/funder, who has granted bioRxiv a license to display the preprint in perpetuity. It is made available under aCC-BY 4.0 International license.

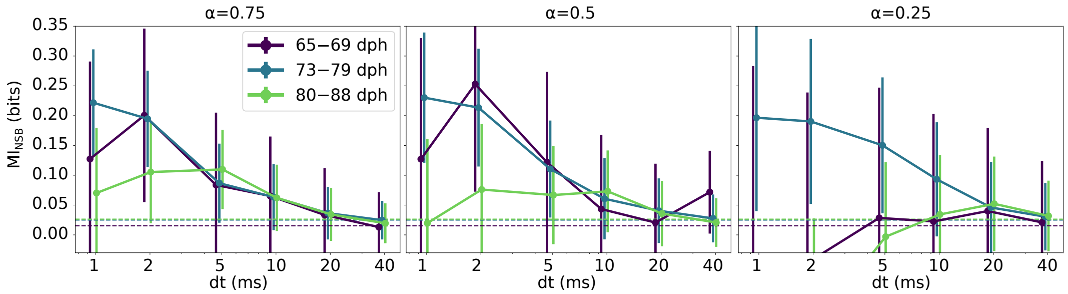

806

Figure 4—figure supplement 1. *Behavior of the mutual information as a function of the data set size supports the hypothesis of the temporally precise neural code.* We plot mutual information estimates as a function of the discretization scale dt of RA activity patterns and the sample set size for different age categories. Each panel correspond to a different fraction α of the full dataset of size N used for mutual information estimation. a) $\alpha = 0.75$, b) $\alpha = 0.5$, c) $\alpha = 0.25$. Below $\alpha = 0.5$, mutual information at high temporal resolution is statistically indistinguishable from zero, suggesting that the evidence of temporally precise neural code in **Figure 4** is not an artifact of sample-size dependent estimation biases.
